# Differences between lung adenocarcinoma and lung squamous cell carcinoma: Driver genes, therapeutic targets, and clinical efficacy

**DOI:** 10.1016/j.gendis.2024.101374

**Published:** 2024-07-11

**Authors:** Yue Shen, Jie-Qi Chen, Xiang-Ping Li

**Affiliations:** a Department of Pharmacy, Xiangya Hospital, Central South University, Changsha, Hunan 410008, China; b National Clinical Research Center for Geriatric Disorders, Xiangya Hospital, Central South University, Changsha, Hunan 410008, China

**Keywords:** Clinical efficacy, Gene mutations, Lung adenocarcinoma (LUAD), Lung squamous cell carcinoma (LUSC), Therapeutic targets

## Abstract

With the rapid advancements in second-generation gene sequencing technologies, a growing number of driver genes and associated therapeutic targets have been unveiled for lung adenocarcinoma (LUAD) and lung squamous cell carcinoma (LUSC). While they are clinically classified as non-small cell lung cancer (NSCLC), they display distinct genomic features and substantial variations in clinical efficacy, underscoring the need for particular attention. Hence, this review provides a comprehensive overview of the latest advancements in driver genes, epigenetic targets, chemotherapy, targeted therapy, and immunotherapy for LUAD and LUSC. Additionally, it delves into the distinctions in signaling pathways and pivotal facets of clinical management specific to these two categories of lung cancer. Moreover, we furnish pertinent details regarding clinical trials pertaining to driver genes and epigenetics, thus establishing a theoretical foundation for the realization of precision treatments for LUAD and LUSC.

## Introduction

Lung cancer is the leading cause of cancer-related deaths worldwide,^1^ broadly classified into non-small cell lung cancer (80%) (NSCLC) and small cell lung cancer (20%). NSCLC can be further categorized into various subtypes, including lung adenocarcinoma (LUAD), lung squamous cell carcinoma (LUSC), adenosquamous carcinoma, large cell carcinoma, sarcomatoid carcinoma, *etc*.^2^ In clinical practice, the two most common pathological types of NSCLC are LUAD (40%) and LUSC (30%).^3^ LUAD originates from the pulmonary epithelium in the lung parenchyma and exhibits distinctive papillary structures and glandular features, while LUSC originates from the basal cells of the lung's respiratory airways and is characterized by keratinized regions and intercellular bridges.^2^ In most prior research and expert guidelines, LUAD and LUSC were often treated with the same chemotherapy regimens. However, following the JMDB study, it was determined that the chemotherapy approach for LUAD differs from that of LUSC.^4^ As precision medicine advances, specific treatments for LUAD and LUSC are rapidly evolving. Numerous clinical studies have revealed significant differences, particularly in the context of targeted therapies, notably epidermal growth factor receptor tyrosine kinase inhibitors (EGFR-TKIs). The distinct driver genes associated with LUAD and LUSC can result in varying prognosis outcomes during clinical targeted therapy. Therefore, a comprehensive understanding of the driver genes for these two types of lung cancer is crucial for clinical practitioners to choose appropriate targeted therapies. Furthermore, the development of specific drugs targeting the significantly expressed gene targets or signaling pathways for both types will bring about substantial breakthroughs in the precision treatment of LUAD and LUSC. Additionally, differences exist between LUAD and LUSC in their immune treatment microenvironment characteristics. This review delineates the treatment targets for LUAD and LUSC into two main categories: common driver gene targets and epigenetic targets. It provides a comprehensive overview of the similarities and differences in treatment targets and clinical approaches, highlighting the promising applications of specific targets in targeted therapy and precision medicine.

## Genomic characteristics of LUAD and LUSC

**Figure 1:**
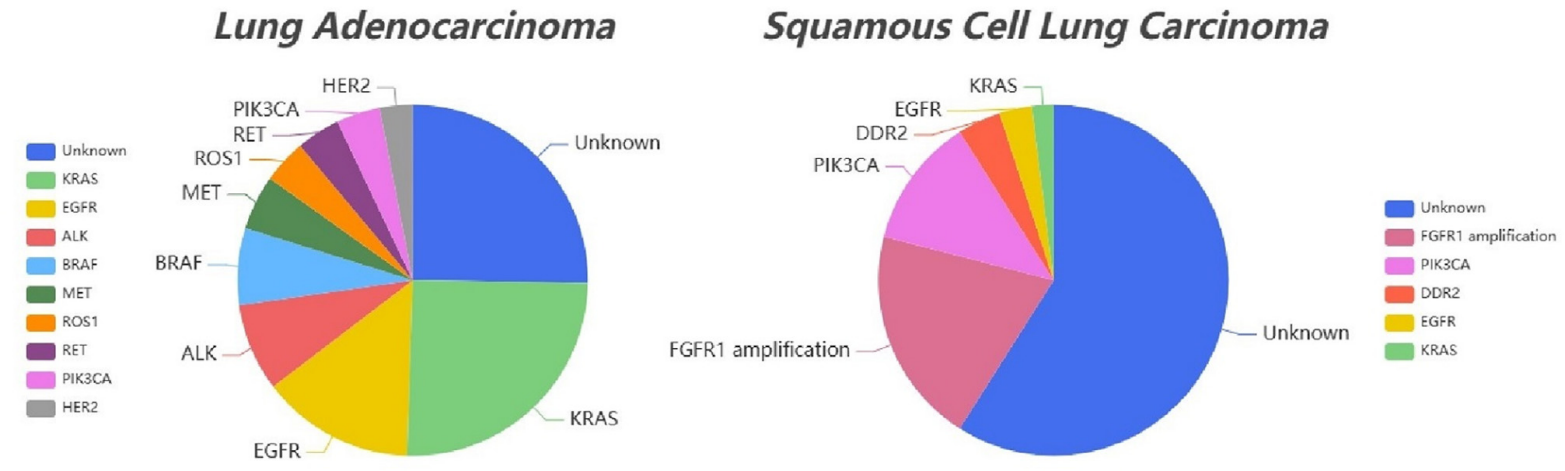
Comparison of targetable driver gene mutation frequencies in lung adenocarcinoma and lung squamous cell carcinoma. Common oncogene mutations in lung adenocarcinoma include *KRAS* (25%), *EGFR* (14%), *ALK* (8%), *BRAF* (7%), *MET* (5%), *ROS1* (5%), *RET* (4%), *HER2* (3%), *etc*. Main oncogene mutations in lung squamous cell carcinoma include *FGFR1* amplification (20%), *PIK3CA* (12%), *DDR2* (3%), *etc*. Genetic mutation data were sourced from The Cancer Genome Atlas database. ALK, anaplastic lymphoma kinase; BRAF, B-Raf proto-oncogene, serine/threonine kinase; DDR2, discoidin domain receptor 2; EGFR, epidermal growth factor receptor; HER2, human epidermal growth factor receptor 2; KRAS, Kirsten rat sarcoma viral oncogene homologue; MET, mesenchymal–epithelial transition; PIK3CA, phosphatidylinositol-4,5-bisphosphate 3-kinase catalytic subunit alpha; ROS1, ROS proto-oncogene 1; RET, rearranged during transfection.

In cancer, somatic copy number alterations are a common genomic abnormality. The differing frequencies of somatic copy number alterations in certain chromosomal regions in LUAD and LUSC^5^^,^^6^ lead to significant differences in their genetic mutations. The oncogenes *KRAS* (Kirsten rat sarcoma viral oncogene homologue), *EGFR*, *BRAF* (B-Raf proto-oncogene, serine/threonine kinase), and *ALK* (anaplastic lymphoma kinase) mutations frequently occur in LUAD, but are rarely found in LUSC (Fig. 1). Conversely, tumor suppressor genes such as *TP53*, *CDKN2A* (cyclin dependent kinase inhibitor 2A), *PTEN* (phosphatase and tensin homolog), and *NOTCH1* (neurogenic locus notch homolog protein 1) exhibit higher mutation frequencies in LUSC. Mutations in these genes lead to frequent alterations in downstream signaling pathways such as PI3K (phosphoinositide 3-kinase)/AKT (protein kinase B)/mTOR (mechanistic target of rapamycin), RAS/RAF/MEK (mitogen-activated extracellular signal-regulated kinase), JAK (Janus kinase)/STAT (signal transducer and activator of transcription), thereby promoting the occurrence and development of both LUAD and LUSC. It is noteworthy that due to the frequent co-occurrence of tumor suppressor gene mutations with other genes, the genomic characteristics of LUAD and LUSC may undergo further alterations.

## Proto-oncogene targets: Directly targetable

### Common proto-oncogene targets in LUAD

#### EGFR

*EGFR*, located on chromosome 7q11.2 in humans,^7^ is a member of the HER (human epidermal growth factor receptor) family. Compared with European patients, Asian LUAD patients exhibit a higher prevalence of *EGFR* gene mutations, reaching around 50%.^8^ These mutations are not limited to changes in DNA sequences, such as exon 19 deletions, but also encompass *EGFR* gene amplifications and elevated EGFR protein expression. It is worth noting that in elderly patients, higher EGFR protein expression levels are observed, whereas there is no such correlation with *EGFR* gene amplification.^9^ Furthermore, this study revealed an association between *EGFR* amplification and a poorer prognosis in LUSC patients. Peng and colleagues confirmed that LUAD patients with *EGFR* amplification often have *TP53* mutations, and *EGFR* amplification is a risk factor for disease progression in patients with brain metastases.^10^ Research indicates that the third-generation TKI drug, osimertinib, can not only reverse resistance to gefitinib and erlotinib in LUAD (T790M mutation) but can also serve as a neoadjuvant therapy, increasing the rate of surgical resection for patients.^11^ Exon 19 deletions and L858R point mutations are the two most common activating mutation types and are often used as biomarkers for TKI-targeted therapy.^12^ For patients with *EGFR* exon 19 deletions and L858R point mutations, osimertinib and gefitinib combined with pemetrexed have demonstrated the longest progression-free survival (PFS).^13^ The limited sample size and prevalent use of non-targeted drugs as first-line treatment in clinical practice for *EGFR*-mutant LUSC patients raise controversy regarding the efficacy of EGFR-TKIs^14^ (Table S1). Currently, there have been four generations of targeted drugs developed for *EGFR* mutations. Among these, the fourth generation, including drugs like TQB3804, U3-1402, and BLU-945, is still in the clinical trial phase.^15^ According to the results published by the American Association for Cancer Research in 2022 and 2023, BLU-945 is one of the fastest-progressing fourth-generation EGFR-TKI drugs, and the successful development of fourth-generation EGFR drugs holds promise in addressing resistance issues associated with third-generation EGFR-TKIs.

#### KRAS

*KRAS* belongs to the RAS oncogene family and is located in the 12q12.1 region.^16^ In contrast to *EGFR* mutations, *KRAS* mutations have a higher prevalence in LUAD patients in Europe, reaching approximately 30%, as opposed to Asian patients.^17^ *KRAS* mutations typically occur in exon 2, 3, or 4 regions.^18^ Among them, the *KRAS G12C* mutation is the most common, accounting for over 50% of *KRAS* mutations. Other *KRAS* mutations include G12V, G12D, and G13C. Due to the complex structure and lack of distinct catalytic activity of KRAS protein 19, targeting KRAS protein has proven to be challenging. Currently, only targeted drugs for the *KRAS G12C* mutation, such as sotorasib and adagrasib, have received approval for market use (Table 1).^20^^,^^21^ When *KRAS*-mutated LUAD is accompanied by LKB1 (liver kinase B1)/STK11 (serine/threonine kinase 11) loss, it can transition to LUSC, a transformation that further leads to resistance to KRAS inhibitors.^22^ This indicates that *KRAS*-mutated LUSC demonstrates a reduced sensitivity to KRAS inhibitors. In addition to directly inhibiting *KRAS* function, promoting the degradation of the KRAS protein can also achieve therapeutic goals, as seen with LC-2,^23^ YN14,^24^ and MS21.^25^ Research by Kostyrko's team at the University of California, USA, has discovered that knocking out *UHRF1* can inhibit the growth of *KRAS*-driven mouse lung cancer tumors, suggesting that *UHRF1* may become a potential therapeutic intervention target for *KRAS*-driven cancers.^26^ However, these drugs and targets are still in the early stages of clinical and preclinical research and require further validation for their safety and effectiveness.

**Table 1:** Targeted therapies for driver genes in lung adenocarcinoma and lung squamous cell carcinoma.

Target	Drug	Phase	Clinical trial	Treatment	Sample	Outcome/status			Sponsor
						mOS (months)	mPFS (months)	ORR (%)	
EGFR	Gefitinib	Listed	NCT00322452	Gefitinib *vs*. carboplatin/paclitaxel	1329	21.6 *vs*. 21.9	9.6 *vs*. 6.3	71.2% *vs*. 47.3%	AstraZeneca
	Erlotinib	Listed	NCT01342965	Erlotinib *vs*. gemcitabine/cisplatin	217	26.3 *vs*. 25.5	11.0 *vs*. 5.5	62.7% *vs*. 33.6%	Hoffmann-La Roche
	Icotinib	Listed	NCT01719536	Icotinib *vs*. pemetrexed/cisplatin	296	NA	11.2 *vs*. 7.9	64.8% *vs*. 33.8%	Betta Pharmaceuticals Co., Ltd.
	Afatinib	Listed	NCT01121393	Afatinib *vs*. gemcitabine/cisplatin	364	22.1 *vs*. 22.2	11.0 *vs*. 5.6	66.9% *vs*. 23.0%	Boehringer Ingelheim
	Dacomitinib	Listed	NCT01774721	Dacomitinib *vs*. gefitinib	452	34.1 *vs*. 27.0	14.7 *vs*. 9.2	74.9% *vs*. 71.6%	Pfizer
	Osimertinib	Listed	NCT02151981	Osimertinib *vs*. pemetrexed + carboplatin/cisplatin	419	26.8 *vs*. 22.5	10.1 *vs*. 4.4	70.6% *vs*. 31.4%	AstraZeneca
	Almonertinib	Listed	NCT02981108	Almonertinib	364	31.5	12.4	65.6%	Jiangsu Hansoh Pharmaceutical Co., Ltd.
	Alflutinib	Listed	NCT03452592	Alflutinib	220	NA	9.6	74.0%	Allist Pharmaceuticals, Inc.
	Amivantamab	Listed	NCT02609776	Amivantamab	780	22.8	8.3	40.0%	Janssen Research & Development, LLC
	Mobocertinib	Listed	NCT02716116	Mobocertinib	334	24	7.3	28.0%	Takeda
KRAS	Sotorasib	Listed	NCT03600883	Sotorasib	126	6.8	12.5	37.1%	Amgen
	Adagrasib	Listed	NCT03785249	Adagrasib	116	12.6	6.5	42.9%	Mirati Therapeutics Inc.
ALK	Crizotinib	Listed	NCT00932893	Crizotinib *vs*. chemotherapy	347	21.7 *vs*. 21.9	7.7 *vs*. 3.0	65% *vs*. 20%	Pfizer
	Alectinib	Listed	NCT02075840	Alectinib *vs*. crizotinib	303	NR	34.8 *vs*. 10.9	82.9% *vs*. 75.5%	Hoffmann-La Roche
	Ceritinib	Listed	NCT01828099	Ceritinib *vs*. chemotherapy	376	NR *vs*. 26.2	16.6 *vs*. 8.1	NA	Novartis Pharmaceuticals
	Brigatinib	Listed	NCT02737501	Brigatinib *vs*. crizotinib	275	NR	24 *vs*. 11	74% *vs*. 62%	Ariad Pharmaceuticals
	Ensartinib	Listed	NCT02767804	Ensartinib *vs*. crizotinib	290	NR	25.8 *vs*. 12.7	74% *vs*. 67%	Xcovery Holding Company, LLC
	Lorlatinib	listed	NCT03052608	Lorlatinib *vs*. crizotinib	296	NR	NR *vs*. 9.3	76% *vs*. 58%	Pfizer
ROS1	Crizotinib	Listed	NCT00585195	Crizotinib	53	51.4	19.2	72.0%	Pfizer
	Entrectinib	Listed	NCT02097810	Entrectinib	161	NR	15.7	67.1%	Hoffmann-La Roche
RET	Selpercatinib	Listed	NCT03157128	Selpercatinib (previously treated) *vs*. selpercatinib (treatment-naive)	356	NR	24.9 *vs*. 22	61% *vs*. 84%	Loxo Oncology, Inc
	Pralsetinib	Listed	NCT03037385	Pralsetinib (previously treated) *vs*. pralsetinib (treatment-naive)	233	NR	16.5 *vs*. 13	NA	Hoffmann-La Roche
MET	Capmatinib	Listed	NCT02414139	Capmatinib (previously treated) *vs*. capmatinib (treatment-naive)	373	NR	5.4 *vs*. 12.4	NR	Novartis Pharmaceuticals
	Tepotinib	Listed	NCT02864992	Tepotinib	337	17.1	8.5	46.0%	EMD Serono Research & Development Institute, Inc.
	Savolitinib	Listed	NCT02897479	Savolitinib	76	12.5	6.8	42.9%	Hutchison Medipharma Limited
	Glumetinib	Listed	NCT04270591	Glumetinib	79	17.3	8.5	65.8%	Haihe Biopharma Co., Ltd.
BRAF	Dabrafenib	Listed	NCT01336634	Dabrafenib *vs*. dabrafenib + trametinib (previously treated BRAF V600E-mutant metastatic NSCLC) *vs*. dabrafenib + trametinib (previously untreated BRAF V600E-mutant metastatic NSCLC)	177	12.7 *vs*. 18.2 *vs*. 17.3	5.4 *vs*. 10.2 *vs*. 10.8	NA	Novartis Pharmaceuticals
HER2	Fam-trastuzumab deruxtecan-nxki	Listed	NCT03505710	Fam-trastuzumab deruxtecan-nxki	91	18.6	8.2	55.0%	AstraZeneca
PIK3CA	Taselisib	Phase II	NCT02785913	Taselisib	31	5.9	2.9	4.8%	SWOG Cancer Research Network
	TOS-358	Phase I	NCT05683418	TOS-358	241	NA	NA	NA	Totus Medicines
	Copanlisib	Phase I	NCT03735628	Copanlisib + nivolumab	16	NA	NA	NA	Bayer
	ASN-003	Phase I	NCT02961283	ASN-003	24	NA	NA	NA	Asana BioSciences
	Buparlisib	Phase I	NCT02128724	Buparlisib + radiotherapy treatment	21	NA	NA	NA	University of Oxford
FGFR1	Nintedanib	Listed	NCT00805194	Docetaxel ± nintedanib	1314	10.1 *vs*. 9.1	3.4 *vs*. 2.7	NA	Boehringer Ingelheim
	Anlotinib	Listed	NCT02388919	Anlotinib *vs*. placebo	439	9.63 *vs*. 6.30	5.37 *vs*. 1.40	9.18% *vs*. 0.70%	Chia Tai Tianqing Pharmaceutical Group Co., Ltd.
	Fexagratinib	Phase II/III	NCT02965378	Fexagratinib	43	7.5	2.7	7.0%	SWOG Cancer Research Network
	Erdafitinib	Phase II	NCT03827850	Erdafitinib	22	NA	NA	NA	Lung Cancer Group Cologne
	Dovitinib	Phase II	NCT01861197	Dovitinib	27	NA	NA	NA	Samsung Medical Center
	AMG479	Phase Ib/II	NCT00807612	AMG 479 + paclitaxel and carboplatin	49	NA	NA	NA	NantCell, Inc.
	BGJ398	Phase I	NCT01004224	BGJ398	208	NA	NA	NA	Novartis Pharmaceuticals
	LY2874455	Phase I	NCT01212107	LY2874455	94	NA	NA	0.0%	Eli Lilly and Company
	Rogaratinib	Phase I	NCT01976741	Rogaratinib	37	NA	2.80	NA	Bayer
	GSK3052230	Phase I	NCT01868022	GSK3052230 + paclitaxel + carboplatin/docetaxel/pemetrexed + cisplatin	65	NA	NA	NA	GlaxoSmithKline
DDR2	∗Dasatinib	Phase II	NCT01491633	Dasatinib	5	NA	NA	NA	Dana-Farber Cancer Institute
STK11	∗Talazoparib	Phase II	NCT04173507	Talazoparib + avelumab	42	7.6	2.7	2%	SWOG Cancer Research Network
	∗TNG260	Phase I/II	NCT05887492	TNG260 *vs*. TNG260 + pembrolizumab	126	NA	NA	NA	Tango Therapeutics, Inc.
	∗Daratumumab	Phase II	NCT05807048	Daratumumab	14	NA	NA	NA	NYU Langone Health
KEAP1	∗Telaglenastat	Phase II	NCT04265534	Pembrolizumab and chemotherapy ± telaglenastat	40	NA	NA	NA	Calithera Biosciences, Inc
	∗Sapanisertib	Phase II	NCT02417701	Sapanisertib (NFEL2 squamous) *vs*. sapanisertib (KEAP1 squamous) *vs*. sapanisertib (KRAS/NFE2L2 or KEAP1 NSCLC)	34	NA	8.9 *vs*. 3.7 *vs*. 2.1	25% *vs*. 16.7% *vs*. 0%	Calithera Biosciences, Inc
TP53	Eprenetapopt	Phase Ib	NCT04383938	Eprenetapopt + pembrolizumab	37	NA	NA	NA	Aprea Therapeutics
	∗Adavosertib	Phase II	NCT02087176	Docetaxel ± adavosertib	48	NA	NA	9.4%	AstraZeneca
	∗Milademetan	Phase II	NCT05012397	Milademetan	65	NA	NA	NA	Rain Oncology Inc
PTEN	∗AZD8186	Phase I	NCT01884285	AZD8186 ± abiraterone acetate/AZD2014	147	NA	NA	NA	AstraZeneca
NOTCH1	∗RO4929097	Phase II	NCT01070927	RO4929097	7	NA	NA	NA	Hoffmann-La Roche
	CB-103	Phase I/II	NCT03422679	CB-103	79	NA	NA	NA	Cellestia Biotech AG
	Crenigacestat	Phase I	NCT02836600	Crenigacestat	12	NA	NA	NA	Eli Lilly and Company
	Brontictuzumab	Phase I	NCT01778439	Brontictuzumab	48	NA	NA	NA	OncoMed Pharmaceuticals, Inc.
CDKN2A	∗Palbociclib	Phase II/III	NCT02785939	Palbociclib	32	7.1	1.7	6.0%	SWOG Cancer Research Network
	∗ABT-348	Phase II	NCT02478320	ABT-348	12	NA	NA	NA	M.D. Anderson Cancer Center
	∗Abemaciclib	Phase II	EUCTR2014-004832-20-DE	Abemaciclib *vs*. docetaxel	150	NA	NA	NA	Eli Lilly & Co.
	∗PF-07248144	Phase I	NCT04606446	PF-07248144 ± fulvestrant/letrozole + palbociclib/PF-07220060 + fulvestrant	186	NA	NA	NA	Pfizer

Notes: The drug information and data in the table were sourced from Pharm Snap, ClinicalTrials.gov, and the EU Clinical Trials Register. Non-direct drugs are indicated by an asterisk (∗). Dasatinib, Bcr-Abl kinase inhibitor, Talazoparib, PARP1/PARP2 inhibitor; TNG260, HDAC1 inhibitor; Daratumumab, CD38 inhibitor; Telaglenastat, glutaminase 1 inhibitor; Sapanisertib, mTOR1/2 inhibitor; Adavosertib, Wee1 inhibitor; Milademetan, MDM2 inhibitor; AZD8186, PI3K inhibitor; RO4929097, γ-secretase inhibitor; Palbociclib, CDK4/6 inhibitor; ABT-348, aurora inhibitor; Abemaciclib, CDK4/6 inhibitor; PF-07248144, KAT6A/B inhibitor; NA, not available; NR, not reached; ORR, objective response rate; mOS, metastatic overall survival; mPFS, metastatic progression-free survival; EGFR, epidermal growth factor receptor; KRAS, Kirsten rat sarcoma viral oncogene homologue; MET, mesenchymal–epithelial transition; RET, rearranged during transfection; HER2, human epidermal growth factor receptor 2; ALK, anaplastic lymphoma kinase; ROS1, ROS proto-oncogene 1; BRAF, B-Raf proto-oncogene, serine/threonine kinase; PIK3CA, phosphatidylinositol-4,5-bisphosphate 3-kinase catalytic subunit alpha; DDR2, discoidin domain receptor 2; FGFR1, fibroblast growth factor receptor 1; STK11, serine/threonine kinase 11; KEAP1, Kelch-like ECH-associated protein 1; PTEN, phosphatase and tensin homolog; NOTCH1, neurogenic locus notch homolog protein 1; CDKN2A, cyclin dependent kinase inhibitor 2A; NSCLC, non-small cell lung cancer.

#### ALK

*ALK* originates from the insulin receptor superfamily.^27^ The most common *ALK* gene mutation is the *EML4* (echinoderm microtubule associated protein like 4)*-ALK* fusion gene, accounting for over 85% of *ALK* mutations.^28^ In young, non-smoking patients with LUAD who do not have *EGFR* mutations, the prevalence of *EML4-ALK* fusion can reach 25%–30%.^29^ *ALK*-positive patients tend to have better treatment responses and survival rates compared with *ALK*-negative NSCLC patients, particularly evident in targeted therapies for *ALK* mutations. Research involving *ALK*-positive non-LUSC patients has shown that those treated with crizotinib achieved a significantly improved PFS of 10.9 months compared with conventional chemotherapy.^30^ Currently, the U.S. Food and Drug Administration (FDA) has approved five targeted drugs for *ALK* mutations, namely crizotinib, ceritinib, alectinib, brigatinib, and lorlatinib. According to the latest research results from the CROWN trial, researchers estimate that the metastatic PFS with the newly approved third-generation ALK inhibitor lorlatinib for treating *ALK*-mutated NSCLC will exceed 60 months.^31^ Lorlatinib remains effective for patients resistant to crizotinib and alectinib,^32^ with a high efficacy rate of up to 82% for patients with brain metastases.^33^

#### ROS1

Similar to the *ALK* gene, *ROS1* (ROS proto-oncogene 1, receptor tyrosine kinase) also belongs to the insulin receptor family.^34^ *ROS1* fusions can be categorized into *CD74* (cluster of differentiation 74)*-ROS1* (44%), *EZR* (Ezrin)*-ROS1* (16%), *SDC4* (syndecan 4)*-ROS1* (14%), and *SLC34A2* (solute carrier family 34 member 2)*-ROS1* (10%).^35^ Due to the 70% amino acid sequence homology in the kinase domain between *ROS1* and *ALK*, patients with *ROS1* gene fusions tend to respond well to most *ALK* inhibitors.^35^^,^^36^ Therefore, patients with *ROS1* rearrangements generally have a favorable prognosis similar to *ALK*-mutated patients. However, it is worth noting that LUAD patients carrying *ROS1* fusion mutations have a higher risk of venous thrombosis.^37^^,^^38^ Currently, both crizotinib and entrectinib have been approved by the FDA for treating advanced NSCLC patients with *ROS1* rearrangements. Unlike crizotinib, entrectinib can penetrate the blood–brain barrier, making it more effective for patients with brain metastases.^39^ However, selective TKIs specifically targeting ROS1 have not yet been developed.

#### RET

*RET* (rearranged during transfection) is one of the members of the calmodulin superfamily, and its mutations primarily occur in untreated, non-smoking young patients.^40^ Patients with *RET* mutations have a higher risk of brain metastasis and pleural dissemination.^41^^,^^42^ Interestingly, tumors with *RET* mutations tend to exhibit a higher sensitivity to regimens that include pemetrexed chemotherapy, possibly due to lower expression levels of thymidylate synthase in *RET*-mutated tumor tissues.^43^^,^^44^ Furthermore, in some studies, *RET* rearrangements have been identified as potential mechanisms of resistance to EGFR-TKIs in *EGFR*-mutated NSCLC.^45^^,^^46^ Currently, there are selective RET inhibitors available for the treatment of LUAD patients with *RET* rearrangements, such as selpercatinib and pralsetinib. Research has shown that selpercatinib and pralsetinib are highly effective in treating *RET*-positive LUAD patients with relatively few side effects.^47^^,^^48^

#### MET

*MET* (mesenchymal–epithelial transition) is located on chromosome 7q31 region.^49^ *MET* exon 14 skipping mutations exhibit some clinical heterogeneity and are commonly found in older, smoking history-associated LUAD patients.^50^^,^^51^ While the occurrence rate of *MET* mutations in LUAD is less than 5%,^52^ it can be as high as around 20% in patients who have shown resistance to molecular targeted therapies such as *EGFR*, *ALK*, and *ROS1*.^53^ *MET* amplification or excessive activation of the MET protein can lead to resistance to first-generation and third-generation EGFR-TKI drugs. However, the use of MET inhibitors has been shown to effectively overcome resistance to third-generation EGFR-TKIs.^54^ For example, in the ORCHARD trial, LUAD patients with *EGFR* and *MET* co-alterations who had become resistant to first-line TKI treatment achieved a disease control rate of 82% with the combination of savolitinib and osimertinib.^55^ Currently, drugs targeting *MET* mutations include crizotinib, savolitinib, capmatinib, and tepotinib. Capmatinib has shown activity against brain metastases as well.^56^

#### BRAF

**Figure 2:**
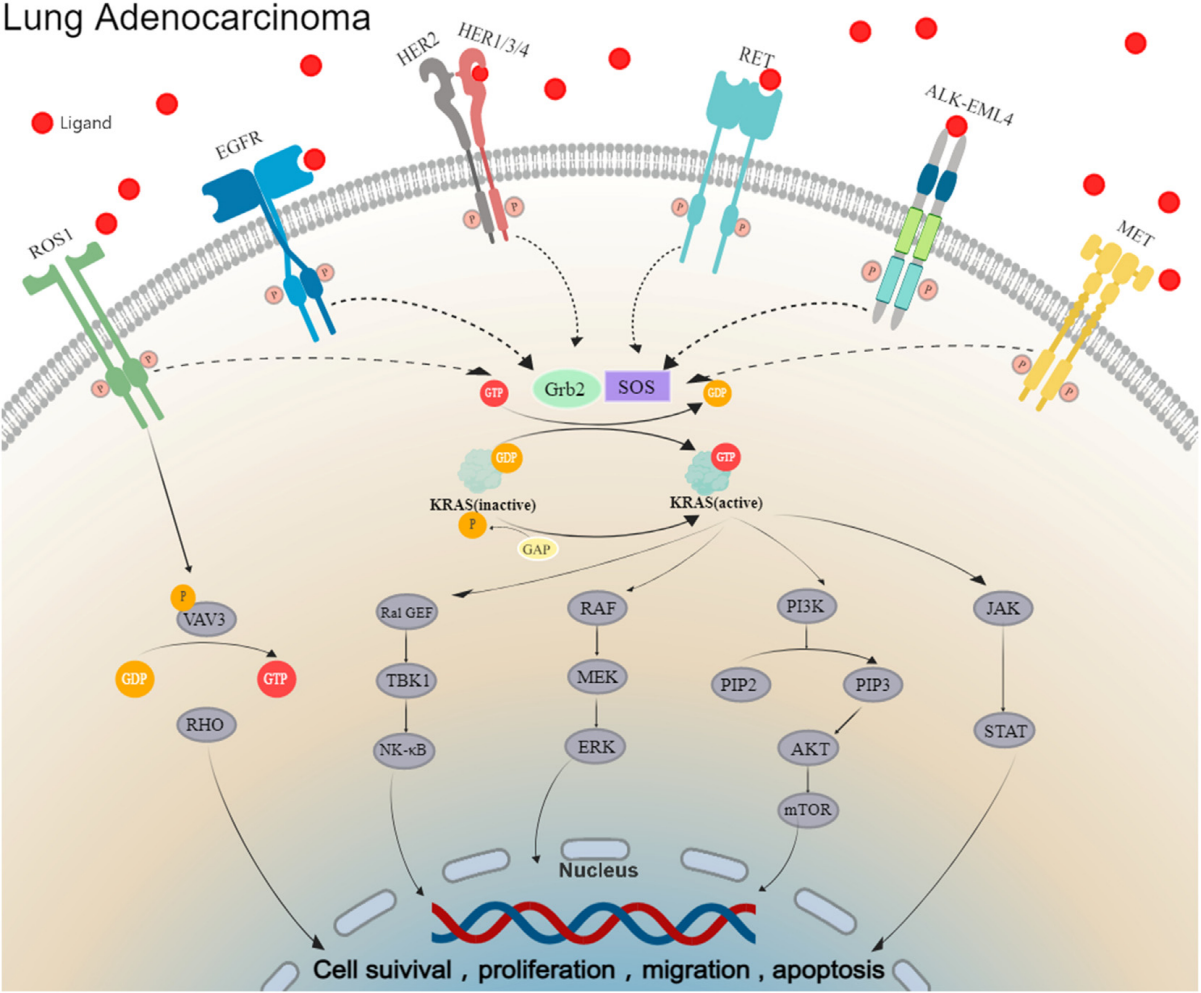
Mechanisms of action of driver genes in lung adenocarcinoma. EGFR, HER2, ALK, ROS1, RET, and MET all belong to the RTK family. Aberrantly activated RTKs bind to Grb2, which recruits SOS proteins to form a complex, catalyzing the binding of KRAS to GTP. Activated KRAS protein can activate downstream signaling pathways including RAF/MEK/ERK, PI3K/AKT/mTOR, JAK/STAT, and Ral-GEF/TBK1/NF-κB, thereby promoting tumor cell survival and proliferation. In addition, ROS1 can activate the VAV3-RHO pathway, further enhancing tumor cell migration and invasion. ALK, anaplastic lymphoma kinase; AKT, protein kinase B; EGFR, epidermal growth factor receptor; EML4, echinoderm microtubule associated protein like 4; ERK, extracellular signal-regulated kinase; GDP, guanosine diphosphate; Grb2, growth factor receptor-bound protein 2; GTP, guanosine triphosphate; HER1/2/3/4, human epidermal growth factor receptor 1/2/3/4; JAK, Janus kinase; KRAS, Kirsten rat sarcoma viral oncogene homologue; MEK, mitogen-activated extracellular signal-regulated kinase; MET, mesenchymal–epithelial transition; mTOR, mechanistic target of rapamycin; NF-κB, nuclear factor-kappa B; PI3K, phosphoinositide 3-kinase; Ral-GEF, Ral guanine nucleotide exchange factors; RET, rearranged during transfection; RHO, rhodopsin, a family member of the small G protein superfamily; ROS1, ROS proto-oncogene 1; RTK, receptor tyrosine kinase; SOS, son of sevenless; STAT, signal transducer and activator of transcription; TBK1, TANK binding kinase 1; VAV3, Vav guanine nucleotide exchange factor 3, a guanine exchange factor for RHO.

*BRAF*, a specific protein kinase composed of 766 amino acids that phosphorylates threonine and serine residues,^57^ is located in the 7q34 region of chromosome 7.^58^ *BRAF V600* mutations account for approximately 30%–50% of all *BRAF* mutations,^59^ and are predominantly found in non-smoking females.^60^ In contrast, non-V600 mutations are mainly observed in male smokers.^61^ *BRAF* mutations lead to reduced response to first-line platinum-based chemotherapy and are associated with poorer prognosis in LUAD patients.^62^ However, studies have indicated that *BRAF* mutations are associated with sensitivity to immune checkpoint inhibitor therapy.^63^, ^64^, ^65^ Because sustained activation of BRAF disrupts the MEK/ERK (extracellular signal-regulated kinase) signaling pathway, leading to excessive cell proliferation and malignancy^66^ (Fig. 2), clinical management of LUAD patients with *BRAF* mutations often involves combination therapy with BRAF and MEK inhibitors. Dual-target treatment with dabrafenib and trametinib has been well-validated for safety and efficacy in various clinical trials^67^^,^^68^ and is currently the only targeted therapy option for *BRAF V600E*-mutated NSCLC in China.

#### HER2

*HER2* (human epidermal growth factor receptor 2) mutations in LUAD primarily involve exon 20 insertion mutations.^69^ This mutation is more common in non-smoking females and Asians^70^ and is associated with a higher likelihood of brain metastasis occurrence compared with other gene mutation statuses.^71^ In the DESTINY-Lung01 trial, trastuzumab deruxtecan demonstrated durable anti-tumor activity (with a median survival of 17.8 months) and a manageable safety profile.^72^ Based on these results, the NCCN guidelines for NSCLC recommend the use of trastuzumab deruxtecan in the treatment of *HER2*-mutated lung cancer.^73^ Furthermore, some HER2-targeted drugs like lapatinib, neratinib, and tucatinib have shown promising efficacy in clinical settings. An EGFR/HER2 dual-targeted inhibitor called pyrotinib has exhibited stronger tumor-suppressive effects in advanced LUAD patients carrying *HER2* exon 20 mutations,^74^ closely related to its comprehensive inhibition of the HER2 signaling pathway.

### Common proto-oncogene targets in LUSC

#### PIK3CA

**Figure 3:**
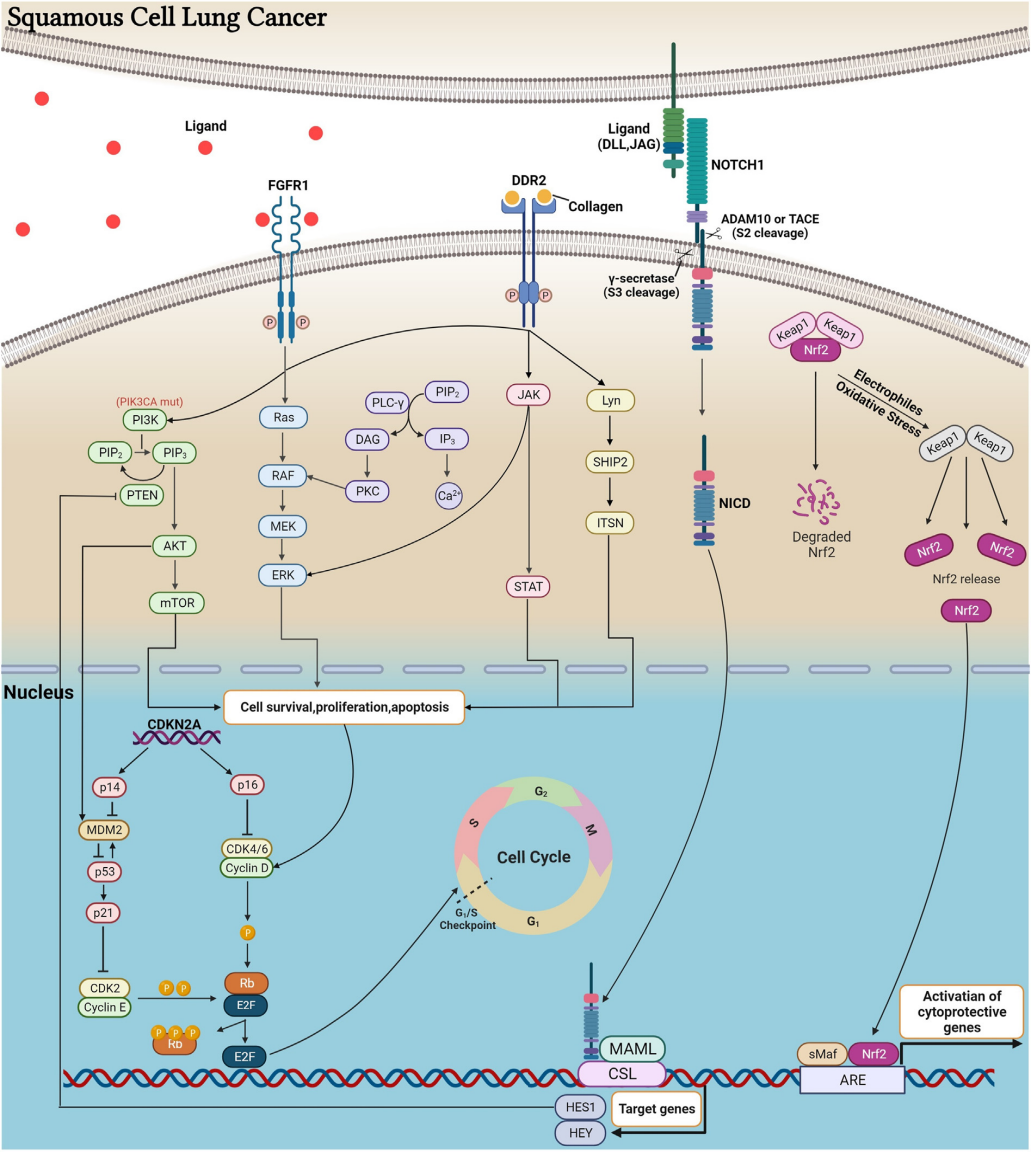
Mechanisms of action of driver genes in lung squamous cell carcinoma. Upon abnormal activation of the FGFR1 receptor, various signaling pathways, including PLCγ/PKC, RAS/RAF/ERK, PI3K/AKT/mTOR, and JAK/STAT, are activated to promote tumor cell growth. Overexpression or mutations in DDR2 can activate the above-mentioned signaling pathways and activate the Lyn/SHIP2/ITSN pathway. The CDK2-CyclinE and CDK4/6-CyclinD complexes, regulated by the *P53* target gene P21, jointly modulate the phosphorylation level of the Rb protein. Hypophosphorylated RB prevents cells from passing through the G1/S checkpoint (also known as the R point), causing cell arrest in the G1 phase. Up-regulation of CyclinD and mutant P53 can lead to the accumulation of unrepaired damaged DNA, ultimately resulting in tumorigenesis. NOTCH1 protein can be activated by binding to NOTCH ligands (Delta-like1/3/4 and Jagged1/2). The Notch receptor is initially cleaved by ADAM10 or TACE and then cleaved by γ-secretase, releasing NICD into the nucleus, where it binds to the transcription factor CSL and recruits MAML protein to form a complex that activates target genes such as *HES* and *HEY*. Among them, *HES1* can inhibit the *PTEN* gene. The product encoded by the *PTEN* gene catalyzes the hydrolysis of PIP3 to PIP2, and its inactivation or mutation leads to activation of the PI3K/AKT pathway. *NRF2*, under steady-state conditions, binds to the protein KEAP1 and is degraded via the proteasome. When exposed to oxidative stress, KEAP1 undergoes a conformational change, releasing NRF2. NRF2 accumulates and translocates to the cell nucleus, where it binds to sMAF protein and activates the transcription of many genes encoding cellular protective factors. Mutations in *KEAP1* lead to the activation of the Nrf2 pathway, promoting the survival of tumor cells. ADAM10, ADAM metallopeptidase domain 10; AKT, protein kinase B; CDK2/4/6, cyclin dependent kinase 2/4/6; CSL, CBF1, suppressor of hairless, lag-1; DDR2, discoidin domain receptor 2; ERK, extracellular signal-regulated kinase; FGFR1, fibroblast growth factor receptor 1; HES1, hairy and enhancer of split 1; ITSN, intersectin; JAK, Janus kinase; KEAP1, Kelch-like ECH-associated protein 1; Lyn, Lck/yes-related protein tyrosine kinase, a member of the Src protein tyrosine kinase family; MAML, mastermind-like; mTOR, mechanistic target of rapamycin; NICD, Notch intracellular domain; NOTCH1, neurogenic locus notch homolog protein 1; NRF2, nuclear factor erythroid 2-related factor 2; PKC, protein kinase C; PI3K, phosphoinositide 3-kinase; PIP2, phosphatidylinositol 4,5-bisphosphate; PIP3, phosphatidylinositol (3,4,5)-trisphosphate; PLCγ, phospholipase C gamma; PTEN, phosphatase and tensin homolog; SHIP2, SH2 domain-containing inositol polyphosphate 5′-phosphatase 2; sMaf, small musculoaponeurotic fibrosarcoma; STAT, signal transducer and activator of transcription; TACE, TNF-α converting enzyme.

*PIK3CA* (phosphatidylinositol-4,5-bisphosphate 3-kinase catalytic subunit alpha), located on chromosome 3, is a representative gene frequently implicated in LUSC, with abnormalities reported in approximately 35% of patients.^75^ Upon occurrence of typical mutations or amplifications in *PIK3CA*, aberrant activation of the PI3K/ATK/mTOR signaling pathway promotes cancer cell proliferation and invasion (Fig. 3). Moreover, *PIK3CA* mutations are associated with chemotherapy resistance and poor prognosis.^76^ In tackling *PIK3CA* mutations, interventions can target not only *PIK3CA* itself but also downstream components of the PI3K signaling pathway, such as AKT and mTOR. Currently, the targeted drug alpelisib for PIK3CA has received approval for breast cancer and has been the subject of numerous clinical trials in the field of LUSC. These trials include PIK3CA inhibitors alpelisib (BYL719), dactolisib (BEZ235), and GDC-0941, mTOR inhibitors everolimus (RAD001) and temsirolimus (CCI-779), and AKT inhibitors miransertib (ARQ-092) and ipatasertib (GDC-0068).

#### *FGFR1* amplification

*FGFR1* amplification is the most common mutation type of FGFR (fibroblast growth factor receptor) in LUSC, accounting for approximately 40%, with an incidence rate of around 20%.^77^ *FGFR1* amplification triggers the PLCγ (phospholipase C gamma)/PKC (protein kinase C), RAS/MAPK (mitogen-activated protein kinase), and PI3K/AKT pathways, promoting angiogenesis as well as the growth and proliferation of tumor cells.^78^ The influence of *FGFR1* amplification on the prognosis for LUSC patients is subject to debate. While one study indicated that *FGFR1* amplification was linked to poorer outcomes,^79^ another found it did not affect PFS and overall survival (OS) in LUSC patients but was strongly associated with lymph node metastasis.^80^ Consequently, whether *FGFR1* amplification can serve as an independent prognostic marker for LUSC requires further research. Currently, various inhibitors targeting FGFR receptors have been developed and are primarily divided into two categories, small molecule TKIs such as AZD4547, BGJ398, and LY2874455, and FGFR antibodies such as FP1039, AMG479, and BIIB022. Unlike the promising results in preclinical models, many FGFR drugs have shown lower disease control rates in clinical trials for lung squamous cell carcinoma, such as AZD4547's phase Ib trial (NCT00979134),^81^ phase II trial (NCT02965378),^82^ rogaratinib's phase II study (NCT01976741),^83^ and BGJ398's phase I trial (NCT01004224).^84^ Therefore, considering the combination of FGFR1 inhibitors with chemotherapy or immunotherapy may hold promise in achieving breakthroughs in anti-tumor activity.

#### DDR2

*DDR2* (discoidin domain receptor 2) is a receptor tyrosine kinase capable of binding to collagen.^85^ Research has shown that phosphorylated *DDR2* in cancer cells can activate the JAK2 (Janus kinase 2)/ERK pathway and may initiate the Ras and PI3K pathways.^86^ In LUSC, about 4% of patients have *DDR2* mutations. These mutations may lead to aberrations in downstream signaling pathways or affect the growth, migration, and invasion of LUSC through the promotion of epithelial–mesenchymal transition.^87^ Studies have indicated that BCR (breakpoint cluster region)-ABL kinase inhibitors used to treat chronic myeloid leukemia, such as dasatinib, nilotinib, and imatinib, have significant inhibitory effects on DDR2 kinase.^88^ In 2011, a phase II clinical study (NCT01491633) investigating the efficacy of dasatinib in advanced squamous cell carcinoma was conducted.^89^ However, because of patients' intolerance to the drug and associated toxicity issues, the trial was terminated prematurely in 2013. Currently, a potential drug targeting *DDR2* mutations in squamous cell carcinoma (4-amino-6-(2,6-dichlorophenyl)-8-methyl-2-(phenylamino)-pyrido [2,3-d] pyrimidin-7(8H)) is in the preclinical trial stage.

## Tumor suppressor gene targets: Difficult to directly target

### Common tumor suppressor gene targets in LUAD

#### STK11/LKB1

*STK11* encodes LKB1 and exhibits a specific high-frequency mutation in LUAD,^90^ often accompanied by *KRAS* mutation.^91^ Inactivation of *STK11* up-regulates the expression of genes associated with angiogenesis and cell migration, thereby enhancing the invasiveness of LUAD.^92^ Despite this, standard treatments such as chemotherapy and immunotherapy tend to be ineffective in patients harboring *STK11* mutations, compounding the challenge in treating such cases. Inactivation of STK11 can lead to aberrant activation of the downstream mTOR signaling pathway and affect glutamine metabolism.^93^ Therefore, mTOR and glutamine inhibitors (such as temsirolimus and everolimus) may have therapeutic effects on it. Furthermore, Shaw et al. found in animal experiments that the combination of the HDAC3 inhibitor entinostat and the MEK inhibitor trametinib effectively inhibits the progression of such tumors.^94^ At present, some non-directly targeted drugs for *STK11* mutations have entered clinical trials (Table 1), but direct targeted drugs for STK11 are still in the research stage. Therefore, improving effective treatment for patients with this mutation remains an urgent clinical challenge.

#### KEAP1

Mutations in *KEAP1* (Kelch-like ECH-associated protein 1) lead to activation of the *Nrf2* (nuclear factor erythroid 2-related factor 2) pathway, thereby promoting tumor cell proliferation and survival (Fig. 2). *KEAP1* mutations are commonly found in LUAD, while *Nrf2* mutations are mainly observed in LUSC. Patients with *KEAP1/NRF2* mutations in LUAD exhibit varying degrees of resistance to chemotherapy, radiotherapy, targeted therapy, and immunotherapy.^95^ Concurrent mutations in *STK11* and *KEAP1* up-regulate the iron death protective gene SCD (stearoyl-CoA desaturase), conferring resistance to drug-induced ferroptosis.^96^ *KEAP1* deficiency also promotes the progression of *KRAS*-driven lung cancer and leads to its dependence on glutamine metabolism.^97^ Research has found that *KEAP1* inactivation results in glucose dependence, rendering lung cancer cells sensitive to glucose inhibitors.^98^ Currently, therapeutic strategies targeting the KEAP/NRF2 signaling pathway primarily focus on glutaminase inhibition, glutamine antagonism, and PI3K-AKT signaling suppression. In preclinical studies, *KEAP1* mutations have shown high selectivity and sensitivity to mTOR inhibitors (such as rapamycin and NVP-BEZ235).^99^ Additionally, clinical trials are underway for the combination therapy of the mTOR inhibitor TAK-228 with the glutaminase inhibitor CB-839 in the treatment of NSCLC.

### Common tumor suppressor gene targets in LUSC

#### TP53

*TP53* mutations are the most prevalent genetic aberrations in both LUAD and LUSC, and their occurrence is closely associated with smoking.^100^^,^^101^ *TP53* mutations lead to impaired cellular apoptosis, DNA damage repair, and cell cycle regulation. Song et al. identified disparities in the *TP53* mutation sites between LUAD and LUSC (Table S1). Furthermore, the detection of *TP53* gene mutations in early-stage LUAD patients often portends a poorer OS, a conclusion not applicable to LUSC.^102^ Some studies suggest that the occurrence of *TP53* mutations in LUSC is related to resistance to platinum-based chemotherapy drugs.^103^^,^^104^ This may be due to alterations in DNA repair mechanisms required for treatment. Besides, immune features associated with *TP53* mutations, such as T cell infiltration and tumor mutation burden, may enhance responsiveness to immunotherapy.^105^ Presently, research on targeted therapies for *TP53* mutations is still in its early stages. For example, APR-246 can restore normal function to mutated *TP53* genes,^106^ while COTI-2 selectively kills tumor cells with *TP53* mutations.^107^ Tumors with *P53* mutations exhibit a heightened dependence on the G2/M checkpoint to maintain genomic stability, rendering them more susceptible to the effects of Wee1 inhibitors such as adavosertib.^108^ Nonetheless, *TP53* mutation is not the most reliable predictive biomarker. This observation may account for the modest benefits observed in many studies when Wee1 inhibitors are combined with chemotherapy. Furthermore, inhibitors targeting *TP53*-protective proteins like Mdm2 and Mdm4 hold promise in restoring TP53 protein function.^109^^,^^110^ Similarly, cell cycle regulatory proteins such as CDK4/6 (cyclin dependent kinase 4/6), which are associated with TP53, may serve as potential therapeutic targets.^111^

#### PTEN

*PTEN* is a major negative regulator of the PI3K/AKT/mTOR signaling pathway. In LUSC, the mutation rate of *PTEN* is approximately 10%, with most mutations occurring in smokers.^112^ This may be related to smoking-induced down-regulation of PTEN expression through immune-mediated mechanisms. Studies have shown that low expression levels of PTEN protein are associated with decreased survival rates in lung cancer patients.^113^ This also underscores the close relationship between down-regulated PTEN protein levels and increased cancer susceptibility. Spoerke et al. discovered that NSCLC models with *PTEN* mutations exhibit high sensitivity to PI3K inhibitors.^114^ Similarly, PTEN inactivation can lead to resistance to PI3K inhibitors.^115^ Furthermore, the imbalance in the expression of PI3K and PTEN can promote immune evasion by tumors.^116^ However, currently, all PI3K/AKT inhibitors or mTOR inhibitors cannot distinguish between LUSC with PTEN loss and those without.

#### NOTCH1

*NOTCH1* is a transmembrane receptor that plays a crucial regulatory role in controlling cell fate, cell proliferation, and differentiation.^117^ Research has revealed that *NOTCH1* has a tumor-suppressive role in LUSC models but promotes tumor growth in LUAD.^118^ According to The Cancer Genome Atlas database, approximately 13% of LUSC patients have *NOTCH1* gene mutations. Furthermore, studies by Yoshida and colleagues found that *NOTCH1* mutations in lung cells are more common among individuals with a history of smoking compared with non-smokers.^119^ Recent research suggests that *NOTCH1* gene mutations may predict the benefit of immunotherapy for LUSC patients.^117^^,^^120^ This may be linked to the activation of DNA damage response pathways and the immune microenvironment associated with *NOTCH1* gene mutations. Since NOTCH1 can inhibit the P53 protein 121, blocking the transmission of the NOTCH1 signaling pathway can increase P53 stability, promoting cell apoptosis. Additionally, interfering with the abnormal activation of NOTCH signaling by inhibiting the key enzyme gamma-secretase is another approach. Some related gamma-secretase inhibitors include PF-03084014, RO4929097, and MK-0752. Furthermore, researchers are exploring antibodies targeting NOTCH1 in both research and clinical trials, such as tarextumab and OMP-52M51.

#### CDKN2A

*CDKN2A* is a tumor suppressor gene located on the short arm of chromosome 9. It encodes the proteins p16INK4a (P16) and p14ARF (P14).^122^^,^^123^ P16 binds to CDK4/6 and cyclin D, inhibiting the transition of the cell cycle from G1 to S phase, thereby affecting cell proliferation.^124^ Research has shown that high expression of P16 in LUSC patients is associated with favorable survival outcomes, although this trend is not observed in LUAD.^125^ CDK inhibitors such as flavopiridol and dinaciclib have demonstrated potential therapeutic effects by inducing cytotoxicity in *CDKN2A*-defective LUSC cells through enhanced apoptosis.^126^ Furthermore, there is a clinical case report of improved efficacy with the CDK4/6 inhibitor abemaciclib in a patient carrying mutations in *CDKN2A*, *PI3K*, and *TP53* simultaneously.^127^ Currently, CDK4/6 inhibitors are primarily used in breast cancer treatment, and research on their use in LUSC is still in the early phases of phase I and phase II clinical trials.

### Emerging driver gene targets: Epigenetic therapeutic targets

The term “epigenetics” was originally coined by Conrad Waddington, encompassing various aspects such as DNA methylation, histone modifications, non-coding RNAs, and chromatin 128. In tumor cells, epigenetic features are extensively dysregulated, driving the development of targeted epigenetic anti-cancer therapies. Regulators associated with DNA methylation and post-translational histone modifications hold promise as emerging driver gene targets in both LUAD and LUSC.^129^ Further studies have shown that downstream effects caused by genetic mutations may lead to different epigenetic modifications in LUAD and LUSC.^92^

### Epigenetic therapy targets for LUAD

#### EZH2

*EZH2* (enhancer of zeste homolog 2), a histone methyltransferase, serves as a critical subunit catalyzing the activity of PRC2 (polycomb repressive complex 2).^130^ Studies have indicated a correlation between elevated EZH2 expression and proliferation and invasion of LUAD cells,^131^ potentially due to its role in regulating the vascular endothelial growth factor-A (VEGF-A) signaling pathway and AKT phosphorylation mechanisms.^132^^,^^133^ Research by Fan and Kim et al. has shown a link between high EZH2 expression in LUAD, smoking, and poor prognosis, whereas there is no significant correlation in LUSC.^134^^,^^135^ It is worth noting that inhibiting EZH2 may lead to resistance to EGFR-TKIs in NSCLC,^136^ and impact the tumor microenvironment, enhancing anti-tumor immunity.^130^ Currently, several EZH2 inhibitors are undergoing clinical and preclinical studies, primarily for lymphoma treatment.^137^ For advanced solid tumors like SHR2554, it is in phase II clinical trials, and the effectiveness of combining EZH2 inhibitors with ICB is under clinical evaluation (Table 2).

**Table 2:** Epigenetic targeted therapies for lung adenocarcinoma and lung squamous cell carcinoma.

Target	Drug	Indication	Phase	Clinical trial	Treatment	Sample	Status	Sponsor
EZH2	SHR-2554	Advanced solid tumor	Phase Ⅰ/Ⅱ	NCT04407741	SHR1701 ± SHR2554	100	Recruiting	Chinese PLA General Hospital
	Tulmimetostat	Advanced solid tumor	Phase Ⅰ/Ⅱ	NCT04104776	Tulmimetostat	213	Recruiting	Constellation Pharmaceuticals
	Tazemetostat	Advanced non-small cell lung cancer	Phase Ⅰ/Ⅱ	NCT05467748	Pembrolizumab + tazemetostat	66	Not yet recruiting	VA Office of Research and Development
	XNW-5004	Advanced solid tumor	Phase I	ChiCTR2100048401	XNW-5004	82	Recruiting	Hematology Hospital of Chinese Academy of Medical Sciences
	TR115	Advanced solid tumor	Phase I	NCT05650580	TR115	26	Recruiting	Tarapeutics Science Inc.
BRD4	RNK05047	Advanced solid tumors	Phase Ⅰ/Ⅱ	NCT05487170	RNK05047	105	Recruiting	Ranok Therapuetics Co. Ltd.
	NHWD-870	NSCLC	Phase I	CTR20202650	NHWD-870	30	Not yet recruiting	Hunan Hengya Pharmaceutical Technology Co Ltd ＆Ningbo Wenda Pharma
	ABBV-075	NSCLC	Phase I	NCT02391480	ABBV-075	128	Completed	AbbVie
	AZD5153	Malignant solid tumors	Phase I	NCT03205176	AZD5153 ± olaparib	49	Completed	AstraZeneca
	PLX2853	Solid tumor	Phase I	NCT03297424	PLX2853	49	Completed	Opna-IO LLC
	SYHA1801	Advanced solid tumors	Phase I	NCT04309968	SYHA1801	186	Recruiting	CSPC ZhongQi Pharmaceutical Technology Co., Ltd.
	HH-3806	Solid tumor	Phase I	ACTRN12622001339741	HH-3806	36	Recruiting	Tigermed Australia Pty Ltd Tigermed Australia Pty Ltd
BET	ZEN-3694	Advanced and refractory solid tumors	Phase Ⅰ/Ⅱ	NCT05053971	ZEN-3694 ± entinostat	30	Recruiting	National Cancer Institute (NCI)
	NUV-868	Advanced solid tumors	Phase Ⅰ/Ⅱ	NCT05252390	NUV-868 ± olaparib/enzalutamide	657	Recruiting	Nuvation Bio Inc.
	ODM-207	Solid tumor	Phase Ⅰ/Ⅱ	NCT03035591	ODM-207	36	Completed	Orion Corporation, Orion Pharma
	Molibresib	Solid tumor	Phase Ⅰ/Ⅱ	EUCTR2014-004982-25-ES	Molibresib	225	Not yet recruiting	GlaxoSmithKline, S.A.
	BI-894999	Advanced solid tumors	Phase I	EUCTR2015-001111-12-BE	BI 894999	158	Not yet recruiting	SCS Boehringer Ingelheim Comm.V
MIR-34a	MRX-34	NSCLC	Phase I	NCT01829971	MRX34	152	Terminated	Mirna Therapeutics, Inc.
HDAC2	HG146	Solid tumor	Phase I	NCT04977167	HG146 ± PD-(L)1	96	Recruiting	HitGen Inc.
	Mocetinostat	Advanced solid tumors	Phase I	NCT00323934	Mocetinostat	42	Completed	Mirati Therapeutics Inc.
	Pracinostat	Advanced solid tumors	Phase I	NCT00741234	Pracinostat	85	Completed	S∗BIO
SOX2	STEMVAC	Stage IV non-squamous non-small cell lung cancer	Phase Ⅱ	NCT05242965	STEMVAC + sargramostim	40	Recruiting	University of Washington
LSD1	Pulrodemstat	Squamous non-small cell lung cancer	Phase Ⅱ	EUCTR2019-004194-95-ES	Pulrodemstat + nivolumab	135	Unknown	Celgene Corporation
	INCB059872	Solid tumors	Phase Ⅰ/Ⅱ	NCT02712905	INCB059872 ± all-trans retinoic acid/azacitidine/nivolumab	116	Terminated	Incyte Corporation
	JBI-802	Locally advanced and metastatic solid tumor	Phase Ⅰ/Ⅱ	NCT05268666	JBI-802	126	Recruiting	Jubilant Therapeutics Inc.
	Baicalin	NSCLC	Phase I	ChiCTR2100051276	Baicalin ± PD-1	152	Recruiting	Beijing Friendship hospital, Capital Medical University
	Pulrodemstat	Relapsed and/or refractory solid tumors	Phase I	NCT02875223	Pulrodemstat + rifampicin/itraconazole	91	Not yet recruiting	Celgene
	Seclidemstat	Advanced solid tumors	Phase I	NCT03895684	Seclidemstat	23	Completed	Salarius Pharmaceuticals, LLC

Notes: The drug information and data in the table were sourced from Pharm Snap, ClinicalTrials.gov, EU Clinical Trials Register, Chinese Clinical Trial Registry. NA, not available; NR, not reached; ORR, objective response rate; OS, overall survival; PFS, progression-free survival; NSCLC, non-small cell lung cancer; PD-L1, programmed cell death ligand 1; PD-1, programmed cell death protein 1; EZH2, enhancer of zeste homolog 2; BRD4, bromodomain containing 4; BET, bromodomain and extraterminal; MIR-34a, microRNA 34a; HDAC2, histone deacetylase 2; SOX2, SRY-box transcription factor 2; LSD1, lysine-specific demethylase 1.

#### BRD4

*BRD4* (bromodomain containing 4) belongs to the BET (bromodomain and extraterminal) protein family, participating in multiple biological processes including transcriptional regulation, DNA damage repair, activation of immune checkpoints, and maintenance of telomeric homeostasis.^138^^,^^139^ Research has shown that inhibiting the activity of BRD4 can modulate the DNA damage response, thereby increasing the sensitivity of cancer cells to stress-inducing agents.^140^ BET inhibitors exert a synergistic effect in combination with homologous recombination defects and PARP (poly(ADP-ribose) polymerase) inhibitors.^141^ Owing to its high expression in LUAD, which correlates with the malignancy of cancer cells and poor prognosis,^142^ BRD4 is considered a potential therapeutic target. Currently, BET protein inhibitors are still in the development and clinical trial stages, including compounds like JQ1, selective BRD2/3/4 inhibitor OTX015 (also known as MK-8628), and novel BET inhibitors like ZEN-3694.^143^ JQ1 demonstrates the potential to overcome cancer cell resistance, such as enhancing sensitivity in platinum-resistant cells and inhibiting the growth of *BRAF*-mutant cancer cells.^144^^,^^145^ In addition to inhibiting the functions of target proteins, Winter et al. also designed a BET protein degrader called dBET1. Treatment with dBET1 leads to the significant destabilization of the BRD4 protein, down-regulation of MYC, and inhibition of proliferation in cancer cells within the tumor.^146^

#### MIR-34a

*MIR-34a* (microRNA 34a) is a tumor-suppressive microRNA molecule, the expression of which is typically significantly reduced in LUAD tissues.^147^ Research indicates that MIR-34a, by targeting EGFR, can inhibit the growth of lung tumors,^148^ and simultaneously regulate the epithelial–mesenchymal transition of tumor cells to suppress tumor metastasis.^149^ This marked reduction in MIR-34a expression often predicts a shorter survival period and holds certain prognostic value.^150^^,^^151^ Furthermore, MIR-34a induces sensitivity of lung cancer cells to cisplatin by modulating the p53/miR-34a/MYCN signaling axis.^152^ Although MIR-34a demonstrates potential therapeutic value, achieving precise gene silencing in drug development is relatively challenging due to miRNAs being endogenously produced. Currently, drugs targeting miRNAs are still in clinical development, with only five drugs undergoing clinical trials. There was once a MIR-34a analogue called “MRX34” initially used for the treatment of solid tumors. Unfortunately, in a phase I clinical trial (NCT01829971), five severe adverse events occurred, with four patients succumbing to them. At last, the drug's development was halted in 2016.

#### HDAC2

*HDAC2* (histone deacetylase 2) is a member of the histone deacetylase family.^153^ Studies have found that HDAC2 is overexpressed in lung cancer tissues. It enhances cancer cell proliferation and invasion by regulating eIF5 (eukaryotic translation initiation factor 5) and eIF6, thus adversely affecting patient prognosis.^154^ Scientists at Harvard Medical School confirmed in a mouse model that combining an HDAC2 inhibitor with a PD-1 (programmed cell death protein 1) antibody significantly suppresses tumor growth and improves survival rates, suggesting that HDAC2 might be a new potential option for combination therapy with PD-1 inhibitors.^155^ Similarly, because HDAC2 can increase the expression of angiogenic factors through its deacetylation activity, inhibiting HDAC2 may reverse this anti-angiogenic drug resistance phenomenon.^156^ Currently, HDAC inhibitors such as vorinostat, romidepsin, belinostat, and panobinostat have received FDA approval for marketing, primarily for lymphomas and myelomas. Chidamide has been approved by the China National Medical Products Administration for the treatment of peripheral T-cell lymphoma and breast cancer. These drugs have brought new hope to cancer treatment, and HDAC2, as an important target, may provide potential options for future cancer therapies.

#### TET

*TET* (ten-eleven translocation enzymes), including *TET1*, *TET2*, and *TET3*, are essential enzymes involved in the regulation of the process of converting 5-methylcytosine on DNA, controlling DNA methylation levels.^157^ Research has shown an association between the up-regulation of TET1 mRNA and the advanced stages of lung cancer.^158^ The study of Qin et al. revealed that TET enzymes suppressed the malignant progression of lung epithelial cells and LUAD by inducing low methylation of key oncogenes in the Wnt signaling pathway.^159^ Furthermore, Wu et al. demonstrated that patients with *TET1* mutations exhibited prolonged PFS and OS when undergoing immunotherapy. This may be closely related to the increased tumor mutational burden caused by *TET1* mutations and the higher presence of infiltrating T lymphocytes in tumors.^160^ Therefore, *TET* mutations can be considered as independent biomarkers for predicting immunotherapy responses in LUAD. Besides, vitamin C, as a critical cofactor, enhances TET enzyme activity, thereby facilitating active DNA demethylation.^161^ However, to determine the optimal dosage and precise clinical efficacy of vitamin C in fully utilizing TET therapy drugs, further large-scale clinical controlled studies are necessary.

### The epigenetic therapeutic targets of LUSC

#### NSD3

*NSD3* (nuclear receptor binding SET domain protein 3) is a histone methyltransferase primarily responsible for catalyzing trimethylation processes on histones.^162^ In 2021, Yuan et al. published significant research finding in *Nature*, revealing the pivotal role of *NSD3* in the development of LUSC. They confirmed in mouse and cell models that the loss of *NSD3* significantly inhibits the growth of LUSC tumors.^163^ Studies also indicate that *NSD3* can interact with *BRD4*, enhancing the sensitivity of lung squamous cell cancer to BET inhibitors.^164^ Currently, there are several NSD3 inhibitors such as BI-9321, MS9715, and SYL2158 that have demonstrated inhibitory effects in lung cancer.^165^ Furthermore, research based on a proteolysis-targeting chimera (PROTAC) strategy has identified small molecule NSD3 degraders (8) capable of effectively reducing NSD3 protein levels in cell models upon single-dose administration.^166^ These findings offer potential directions for clinical treatment of LUSC.

#### SOX2

*SOX2* (SRY-box transcription factor 2) is a pluripotent transcription factor involved in regulating cellular self-renewal and differentiation.^167^ Preclinical studies have shown that inhibiting tumor growth in LUSC cell lines can be achieved through RNA interference targeting SOX2.^168^ Wilbertz et al. have also identified low-level amplification of SOX2 in 68% of LUSC, distinct from LUAD. Interestingly, high expression of SOX2 in LUSC is associated with increased patient survival.^169^ This could be attributed to the fact that SOX2 overexpression leads to significant differentiation of squamous cells,^170^ thus providing better prognostic outcomes for LUSC patients. Due to the absence of an active site for small molecule inhibitors targeting SOX2, specific drugs aimed at SOX2 are not currently available for clinical use. Consequently, current research primarily focuses on understanding the regulation mechanisms and biological functions of SOX2. Some studies suggest that LUSC cell lines with *SOX2* amplification may exhibit dependencies on EZH2 and associations with LSD1 (lysine-specific demethylase 1).^171^ Therefore, potential therapeutic strategies targeting EZH2 and LSD1 could be considered for treating this subset of LUSC.

#### LSD1

*LSD1* is a lysine-specific demethylase primarily involved in the demethylation of di-methylated/mono-methylated histone H3 lysine 4.^172^ Inhibiting LSD1 can promote cell differentiation, reactivate the expression of tumor suppressor genes, and effectively control tumor development.^173^ Studies indicate that in LUSC expressing SOX2, the levels of LSD1 are significantly elevated, and LSD1 inhibitors exhibit a high degree of selectivity against them.^174^ Furthermore, LSD1 inhibitors can enhance the expression of pro-inflammatory cytokines in Treg cells and facilitate CD8^+^ T cell infiltration, converting “cold tumors” into “hot tumors” in immunotherapy. This enhances tumor cell sensitivity to immune checkpoint inhibitors.^175^ Currently, numerous LSD1 inhibitors have entered clinical trials, including ORY-1001, ORY-2001, INCB059872, IMG-7289, and CC-90011.^176^ However, most of these trials are focused on hematological malignancies, and further research is needed to assess their clinical efficacy in solid tumors.

#### SETD8

*SETD8* (SET domain-containing protein 8), also known as histone H4 lysine 20 methyltransferase, primarily functions in the mono-methylation modification of lysine 20 on histone H4.^177^ Additionally, *SETD8* is involved in the repair of DNA double-strand breaks through the c-NHEJ (canonical non-homologous end joining) pathway,^178^ and interacts with TWIST, enhancing the invasive capabilities of tumors.^179^ Research has shown that miR-502 can regulate the expression of SETD8 protein, thereby promoting the survival of lung cancer patients.^180^ One study demonstrated that inhibiting SETD8 could suppress the growth of LUSC and enhance its sensitivity to chemotherapy drugs.^181^ Furthermore, another study indicated that SETD8 inhibitors could inhibit tumor angiogenesis.^182^ However, now only a few SETD8 inhibitors have cellular activity, such as NSC663284, BVT948, and ryuvidine.^183^ Accordingly, there is an urgent need for improving the structure of these lead compounds and designing a new generation of selective and efficient SETD8 inhibitors.

#### KMT2D

*KMT2D* (lysine methyltransferase 2D), also known as *MLL2* (mixed lineage leukemia 2), is primarily responsible for catalyzing amino methylation of histone H3 lysine 4.^184^ Gene mutations or deletions of *KMT2D* occur in approximately 20% of LUSC patients.^185^ Research by Wong and colleagues, published in *Cancer Cell*, demonstrates *KMT2D* as a crucial epigenetic target in LUSC tumorigenesis.^186^ Through mouse models and patient-derived xenograft experiments, they discovered that loss of *KMT2D* made LUSC more sensitive to RTK (receptor tyrosine kinase)-RAS inhibitors. Additionally, LUSC patients with *KMT2*D mutations typically exhibit lower rates of recurrence-free survival.^187^ The loss of *KMT2D* extensively impairs gene signaling in super-enhancers, suppresses glycolysis, and reduces the levels of acetylated histone H3 lysine 27.^188^ This discovery opens up new therapeutic strategies for *KMT2D*-deficient lung cancer, such as using glycolysis inhibitors. Moreover, since the loss of *KMT2D* leads to overactivation of MEK in the RAS/MAPK pathway,^189^ MAPK pathway inhibitors can also be considered potential drugs for treating *KMT2D* mutations.

### Clinical treatment disparities

**Figure 4:**
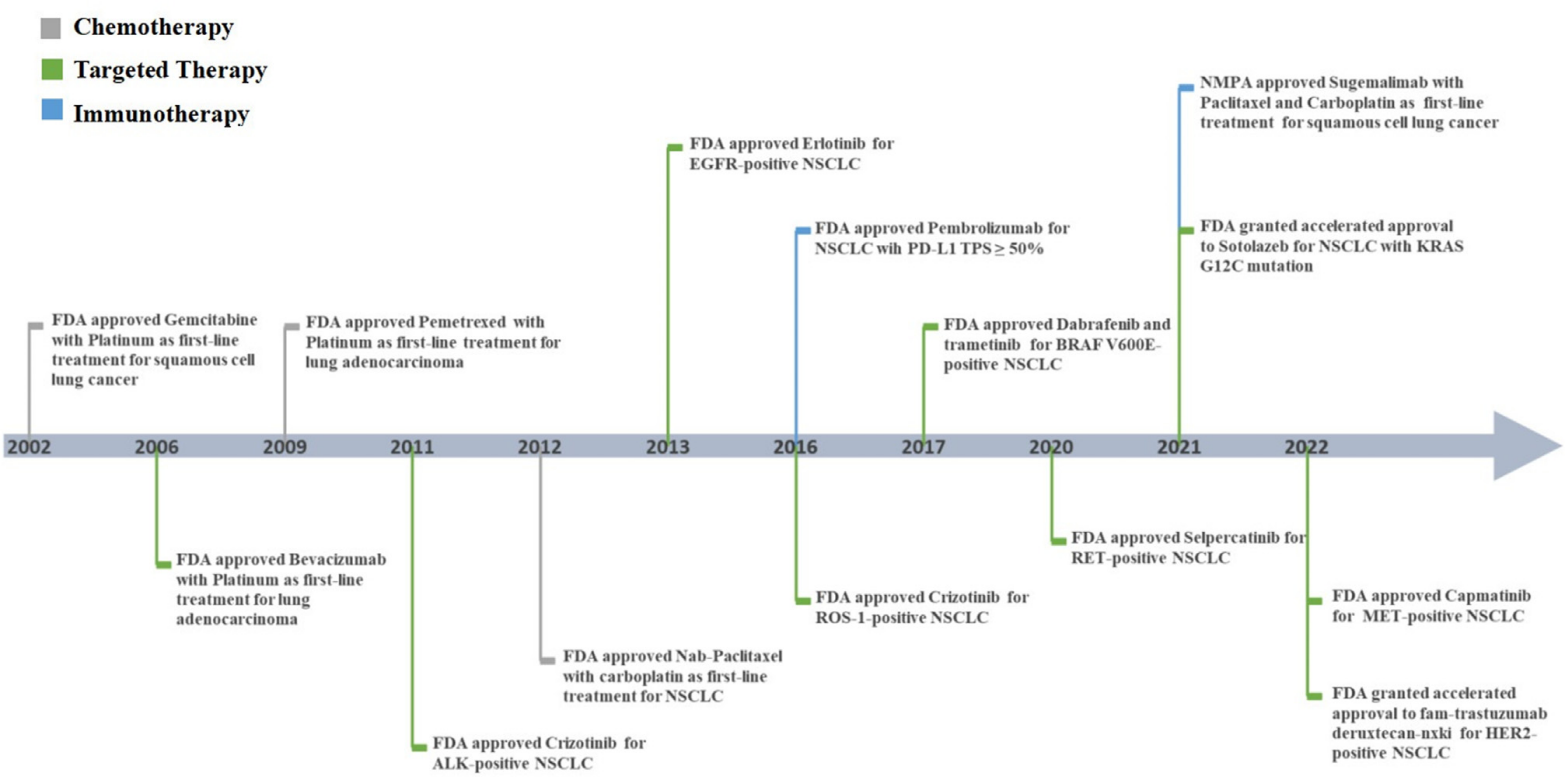
Clinical treatment timeline for lung adenocarcinoma and squamous cell carcinoma. The emergence of EGFR-TKI drugs shifted lung adenocarcinoma from conventional chemotherapy to targeted therapy. However, over the past decade, no targeted therapies have been introduced for lung squamous cell carcinoma. With the advent of the immunotherapy era, the available treatment options for squamous cell carcinoma have gradually expanded. EGFR, epidermal growth factor receptor; HER2, human epidermal growth factor receptor 2; KRAS, Kirsten rat sarcoma viral oncogene homologue; MET, mesenchymal–epithelial transition; NSCLC, non-small cell lung cancer; PD-L1, programmed cell death ligand 1; RET, rearranged during transfection; TKI, tyrosine kinase inhibitor.

Diverse origins of cells, high-risk factors such as tobacco exposure, and variations in driver genes largely determine the distinct pathogenic mechanisms between LUAD and LUSC. In the processes of chemotherapy, targeted therapy and immunotherapy, and distinct strategies and protocols for the clinical management of LUAD and LUSC are gradually emerging (Fig. 4).

### Chemotherapy

Pemetrexed is a chemotherapy drug that targets thymidylate synthase and plays a significant role in first-line treatment for LUAD (Table S2). However, in a large phase III clinical trial that was double-blind and placebo-controlled, it was found that when pemetrexed was used in combination therapy for LUSC, there was no significant difference in PFS and OS between the two groups of patients.^190^ This difference may be attributed to the high expression of thymidylate synthase in LUSC, which reduces patients' sensitivity to folate-based drugs. Additionally, JMDB research also found that when combining pemetrexed with platinum-based drugs for treating LUAD patients, there was a significant extension in OS, surpassing the combination of gemcitabine and platinum-based drugs. Conversely, in LUSC, the combination of gemcitabine and platinum-based drugs led to a relatively longer OS, demonstrating better efficacy compared with the combination of pemetrexed and platinum-based drugs.^4^ The CTONG1002 study further compared the clinical efficacy of albumin-bound paclitaxel/carboplatin with gemcitabine/carboplatin and found that the albumin-bound paclitaxel group had a superior improvement in quality of life compared with the gemcitabine group.

### Targeted therapy

Compared with LUAD, LUSC has fewer driver gene targets, which limits the effectiveness of targeted therapy. However, in the SQUIRE trial, the combination of necitumumab with gemcitabine and cisplatin significantly improved the OS of late-stage LUSC patients. Consequently, necitumumab has been approved for targeted therapy in LUSC with EGFR mutations.^191^ Bevacizumab is a monoclonal antibody targeting vascular endothelial growth factor (VEGF), but it may increase the risk of pulmonary hemorrhage. Due to the central location and susceptibility to bleeding often observed in LUSC patients, bevacizumab is contraindicated in this subgroup. For advanced non-squamous NSCLC patients, as indicated by findings from the ARIES study, both adenocarcinoma and non-adenocarcinoma patients can benefit from bevacizumab treatment.^192^ Moreover, adenocarcinoma patients showed slightly higher OS and PFS compared with non-adenocarcinoma patients. Unless contraindicated, the use of bevacizumab is advantageous for LUAD patients.

### Immunotherapy

At present, first-line treatments for both LUAD and LUSC involve immunotherapy drugs. In comparison to adenocarcinoma, LUSC more commonly expresses PD-L1 (programmed cell death ligand 1), and immune cell infiltration, including macrophages, is more pronounced.^193^ LUSC exhibits a higher somatic mutation frequency and stronger immunogenicity, offering opportunities for the treatment of advanced-stage patients. Based on studies such as KEYNOTE-024, KEYNOTE-042, and KEYNOTE-407,^194^, ^195^, ^196^ the 2020 CSCO (Chinese Society of Clinical Oncology) guidelines recommended the use of pembrolizumab for first-line treatment of advanced LUSC. Currently, there is a broader selection of immunotherapies for LUSC. In a phase III clinical trial, GEMSTONE-302, which included both LUSC and LUAD, it was found that the addition of avelumab to chemotherapy showed superiority in treating LUSC (hazard ratio: 0.59 *vs*. 0.34),^197^ Furthermore, on October 31, 2022, toripalimab gained approval for first-line treatment in combination with carboplatin and albumin-bound paclitaxel for locally advanced or metastatic squamous NSCLC patients. Similarly, the domestically developed immune drug camrelizumab in combination with chemotherapy is recommended in the 2022 CSCO guidelines for first-line treatment of advanced LUSC.

## Conclusion and prospects

LUSC and LUAD, two subtypes of NSCLC, exhibit numerous differences in driver genes, treatment targets, and clinical efficacy. In general, gene mutations in LUAD, such as *EGFR*, *MET*, and *BRAF* mutations, often occur on chromosome 7, while gene mutations in LUSC, such as *PIK3CA*, *SOX2*, and *TP63*, are frequently observed on chromosome 3. LUAD exhibits a higher frequency of proto-oncogene mutations, particularly mutations in receptor tyrosine kinases such as EGFR, ALK, ROS, RET, MET, and HER2. In contrast, LUSC demonstrates widespread inactivation of tumor suppressor genes such as *TP53*, *KEAP1*, *PTEN*, and *CDKN2A*, along with fewer directly targeted driver genes, characteristic of a multi-driver mutation profile. Although high-frequency gene mutations in LUAD, such as *EGFR* and *KRAS*, can also be found in LUSC, their mutation frequencies are all below 5%.^198^ Additionally, EGFR mutations in LUSC are often not the classic sensitive mutations, leading to suboptimal responses when patients are treated with EGFR-TKIs.^199^ Beyond differences in gene mutation frequencies, the roles of the same genes (*e.g.NOTCH1*) may be entirely opposite in LUAD and LUSC (Table S1). However, such distinctions are not fully reflected in guidelines both domestically and internationally. Extensive clinical practice has shown that LUAD patients may undergo adenosquamous transformation following chemotherapy, targeted therapy, and immunotherapy.^200^ This suggests that adenosquamous transformation may represent a potential mechanism for the development of resistance in clinical lung cancer patients.

As one of the most crucial tumor suppressor genes in humans, *TP53* carries profound clinical importance for the development of targeted therapies. Mutations in *TP53* increase chromosomal instability in tumor cells, further leading to the amplification of oncogenes and loss of tumor suppressor genes.^123^ Therefore, besides focusing on directly targeted genes, greater emphasis should be placed on therapeutic strategies aimed at mutations in tumor suppressor genes. Despite facing challenges like drug resistance, discrepancies in animal models, and the constraints of using single-agent TP53 therapy,^201^ no TP53-targeted drugs have yet received approval. Nonetheless, we unanimously agree that further exploration of the biological characteristics of TP53 is highly necessary.

The discovery of epigenetic therapy targets in LUAD and LUSC, exemplified by *EZH2* and *NSD3*, has further advanced the development of precision medicine. However, it also brings about some new challenges, such as the lack of small molecule binding sites that are difficult to drug, limited cellular activity inhibitory compounds, and potential toxicity issues. Emerging therapeutic approaches, such as the introduction of PROTACs,^202^ seem to offer new hope for these challenging druggable targets. Additionally, PROTACs, by virtue of their protein degradation mechanism, hold the potential to overcome the issue of small molecule drug resistance. The improvement of lead compound structures through computer molecular screening techniques may contribute to enhancing the anti-tumor activity and safety of such drugs.

Since the introduction of the first-generation EGFR-TKI drug gefitinib in the year 2000, there has been a profound transformation in the treatment landscape for LUAD and LUSC. The highly complex genomic landscape and carcinogenic pathways of LUSC contribute to its elevated mutational burden,^123^ posing challenges in elucidating its true driver genes. The recurrent molecular alterations in LUSC make it challenging to establish representative mouse models, greatly limiting the development and application of targeted therapies. Additionally, the higher proportion of driver gene amplifications in LUSC often results in “cross-reactivity” of PIK3CA inhibitors and FGFR1 inhibitors with wild-type genes, leading to significant toxicity reactions in clinical trials. Due to the poor selectivity of biomarkers for targeted therapy in LUSC,^203^ this further exacerbates the dilemma of balancing moderate benefits and high toxicity in targeted therapy for LUSC. Treatment options for LUSC are fewer compared with LUAD, and patients with advanced LUSC often have a poorer prognosis. Developing multi-targeted inhibitors targeting the characteristic multiple driver mutations of LUSC may lead to greater breakthroughs in its treatment. Owing to the specificity of tumor suppressor genes, the landscape of targeted therapy for LUSC may further focus on epigenetic therapy. The emergence of immune drugs such as PD-1/L1 inhibitors has revolutionized the treatment of LUSC, significantly improving patient survival prognosis. Therefore, future research may need to stratify management among patients treated with frontline targeted and immune therapies to determine the priority of targeted drugs.

Currently, targeting epigenetic modifications has become a crucial strategy in overcoming resistance to chemotherapy in cancer. The interplay between immunotherapy and epigenetic processes has facilitated the combination of epigenetic therapy and immunotherapy. For instance, LSD1 inhibitor enhances the immunogenicity of tumors, activates T-cell immune activity, and augments the effectiveness of immune checkpoint inhibitors.^175^ Additionally, the inactivation of relevant oncogenic pathways in LUSC can influence the tumor's immune microenvironment. For instance, the PI3KCA inhibitor AMG319 can lead to the activation of CD4^+^ and CD8^+^ T-cell responses and a weakening of the function of tumor-infiltrating Tregs.^204^ Epigenetic drugs appear to synergize with targeted therapies as well, such as the combination of histone deacetylase inhibitors with TKIs, which can restore sensitivity to targeted treatments.^205^ This provides a new perspective for addressing the current resistance to TKI drugs in LUAD. Based on the molecular biological characteristics of the two subtypes of lung cancer, a rational combination of epigenetic therapy, targeted therapy, and immunotherapy may achieve more effective treatment. The genetic characteristics of LUAD and LUSC allow patients to be molecularly classified based on histological classification, which will facilitate the formulation of personalized treatment plans, thereby leading to better treatment outcomes and clinical prognosis.

## Conflict of interests

All authors declared no conflict of interests related to this work.
