# Supplementary material for: Differences between lung adenocarcinoma and lung squamous cell carcinoma: Driver genes, therapeutic targets, and clinical efficacy
Source: Genes Dis. 2024 Jul 11;12(3):101374. doi: 10.1016/j.gendis.2024.101374 (PMC11904499; doi:10.1016/j.gendis.2024.101374)
Supplement: Multimedia component 1 [file mmc1.docx]

**Table S1 Differences in the Same Target Genes Between LUAD and LUSC**

| **Type**  **Genes** | **LUAD** | **LUSC** |
| --- | --- | --- |
| **EGFR^1^** | **Mutation Rate:** 14% | **Mutation Rate:** 3% |
|  | **Mutation Subtype:** Some common sensitizing mutations include deletion in exon 19 and L858R substitution in exon 21. | **Mutation Subtype:** Sensitizing mutations are rare. |
|  | **Clinical Treatment:** Common EGFR-TKIs are indicated for LUAD. | **Clinical Treatment:** Afatinib is currently the only EGFR TKI approved for LUSC as a single agent. |
| **KRAS^2,3^** | **Mutation Rate:** 25% | **Mutation Rate:** 2% |
|  | **Mutation Subtype:** G12C>G12V>G12D>G13C | **Mutation Subtype:** G12C>G12D>G13C>G12V |
|  | **Mutation characteristics:**  The frequency of KRAS mutations in LUAD appears to constant across tumour grades. | |
| **ALK** | **Mutation Rate:** 8% | **Mutation Rate:** 4% |
| **BRAF^4^** | **Mutation Rate:** 7% | **Mutation Rate:** 3% |
|  | **Mutation characteristics:**  The majority of BRAF mutations in LUAD belong to the functional categories (oncogenic or likely oncogenic). | **Mutation characteristics:**  ①More than two-thirds of BRAF mutations in LUSC belong to the unknown category.  ②High BRAF expression is correlated with poor relapse-free survival (RFS) in LUSC. |
| **PIK3CA^5^** | **Mutation Rate:** 4% | **Mutation Rate:** 12% |
|  | **Mutation characteristics:**  The most common co-occurring driver genes with PIK3CA are TP53 and ERBB2. | **Mutation characteristics:**  The most common co-occurring driver genes with PIK3CA are TP53 and CDKN2A. |
| **FGFR1 ^6^** | FGFR1 amplifications are most commonly encountered in LUSC and only rarely in LUAD. | |
| **STK11** | **Mutation Rate:** 12% | **Mutation Rate:** 1% |
| **KEAP1^7^** | **Mutation Rate:** 17% | **Mutation Rate:** 10% |
|  | **Mutation characteristics:** KEAP1 mutations correlate with increased PD-L1 expression in LUAD, while no obvious correlation is observed in LUSC. | |
| **TP53^8^** | **Mutation Rate:** 50% | **Mutation Rate:** 83% |
|  | **Mutation sites：** T125T/X125-splice mutations are common in LUAD; R158G/H/L mutations prevalent in LUSC. | |
|  | **Mutation characteristics:**  TP53 mutations correlate with a worse OS in Early-stage LUAD , but they are not associated with OS in LUSC. | |
| **PTEN** | **Mutation Rate:** 2% | **Mutation Rate:** 11% |
| **NOTCH1^9^** | **Mutation Rate:** 4% | **Mutation Rate:** 8% |
|  | **Mutation characteristics:**  NOTCH1 promotes tumor growth in LUAD but suppresses it in LUSC. | |
| **CDKN2A^10,11^** | **Mutation Rate:** 5% | **Mutation Rate:** 14% |
|  | **Mutation characteristics:**  Homozygous deletion (HD) of CDKN2A is associated with poor outcomes in LUAD. | **Mutation characteristics:**  CDKN2A mutations can up-regulate the expression of immune response in early-stage LUSC. |

**References:**

1. Lau, S.C.M., Pan, Y., Velcheti, V., and Wong, K.K. (2022). Squamous cell lung cancer: Current landscape and future therapeutic options. Cancer Cell *40*, 1279-1293. 10.1016/j.ccell.2022.09.018.

2. Acker, F., Stratmann, J., Aspacher, L., Nguyen, N.T.T., Wagner, S., Serve, H., Wild, P.J., and Sebastian, M. (2021). KRAS Mutations in Squamous Cell Carcinomas of the Lung. Front Oncol *11*, 788084. 10.3389/fonc.2021.788084.

3. Herbst, R.S., Morgensztern, D., and Boshoff, C. (2018). The biology and management of non-small cell lung cancer. Nature *553*, 446-454. 10.1038/nature25183.

4. Yi, Q., Peng, J., Xu, Z., Liang, Q., Cai, Y., Peng, B., He, Q., and Yan, Y. (2022). Spectrum of BRAF Aberrations and Its Potential Clinical Implications: Insights From Integrative Pan-Cancer Analysis. Front Bioeng Biotechnol *10*, 806851. 10.3389/fbioe.2022.806851.

5. Huang, Q., Zhou, Y., Wang, B., Zhao, Y., Zhang, F., and Ding, B. (2022). Mutational landscape of pan-cancer patients with PIK3CA alterations in Chinese population. BMC Med Genomics *15*, 146. 10.1186/s12920-022-01297-7.

6. Savari, O., Febres-Aldana, C., Chang, J.C., Fanaroff, R.E., Ventura, K., Bodd, F., Paik, P., Vundavalli, M., Saqi, A., Askin, F.B., et al. (2023). Non-small cell lung carcinomas with diffuse coexpression of TTF1 and p40: clinicopathological and genomic features of 14 rare biphenotypic tumours. Histopathology *82*, 242-253. 10.1111/his.14801.

7. Xu, X., Yang, Y., Liu, X., Cao, N., Zhang, P., Zhao, S., Chen, D., Li, L., He, Y., Dong, X., et al. (2020). NFE2L2/KEAP1 Mutations Correlate with Higher Tumor Mutational Burden Value/PD-L1 Expression and Potentiate Improved Clinical Outcome with Immunotherapy. Oncologist *25*, e955-e963. 10.1634/theoncologist.2019-0885.

8. Xu, S., Wang, Y., Ren, F., Li, X., Ren, D., Dong, M., Chen, G., Song, Z., and Chen, J. (2020). Impact of genetic alterations on outcomes of patients with stage I nonsmall cell lung cancer: An analysis of the cancer genome atlas data. Cancer Med *9*, 7686-7694. 10.1002/cam4.3403.

9. Sinicropi-Yao, S.L., Amann, J.M., Lopez, D.L.Y., Cerciello, F., Coombes, K.R., and Carbone, D.P. (2019). Co-Expression Analysis Reveals Mechanisms Underlying the Varied Roles of NOTCH1 in NSCLC. J Thorac Oncol *14*, 223-236. 10.1016/j.jtho.2018.10.162.

10. Peng, Y., Chen, Y., Song, M., Zhang, X., Li, P., Yu, X., Huang, Y., Zhang, N., Ji, L., Xia, L., et al. (2022). Co-occurrence of CDKN2A/B and IFN-I homozygous deletions correlates with an immunosuppressive phenotype and poor prognosis in lung adenocarcinoma. Mol Oncol *16*, 1746-1760. 10.1002/1878-0261.13206.

11. Choi, M., Kadara, H., Zhang, J., Parra, E.R., Rodriguez-Canales, J., Gaffney, S.G., Zhao, Z., Behrens, C., Fujimoto, J., Chow, C., et al. (2017). Mutation profiles in early-stage lung squamous cell carcinoma with clinical follow-up and correlation with markers of immune function. Ann Oncol *28*, 83-89. 10.1093/annonc/mdw437.

**Table S2 First-line Treatment Strategies for LUAD and LUSC (Stage IV)**

| **Type First-line Treatment** | **Performance Status (PS)** | **Lung Adenocarcinoma** | **Squamous Cell Lung Cancer** | **PD-L1expression** |
| --- | --- | --- | --- | --- |
| **Chemotherapy** | PS=0~1 | - NCCN：   **Contraindications to PD-1 /PD-L1 inhibitors:**  Carboplatin+Albumin-bound paclitaxel/  Paclitaxel/Docetaxel/Etoposide/Gemcitabine/  Pemetrexed  OR  Cisplatin+Docetaxel/Etoposide/Gemcitabine/  Paclitaxel/ Pemetrexed  OR  Gemcitabine+Docetaxel /Vinorelbine   - CSCO：   Pemetrexed in combination with Carboplatin  /Cisplatin + Pemetrexed monotherapy maintenance treatment  OR  Carboplatin/Cisplatin +Gemcitabine/Docetaxel  /Paclitaxel/liposomes Paclitaxel /Vinorelbine/  Pemetrexed/Paclitaxel polymer micelles | - NCCN：   **Contraindications to PD-1 /PD-L1inhibitors:**  Carboplatin+Albumin-bound paclitaxel /Docetaxel /Gemcitabine/Paclitaxel  OR  Cisplatin+Docetaxel/Etoposide/Gemcitabine /Paclitaxel  OR  Gemcitabine+Docetaxel /Vinorelbine   - CSCO：   Carboplatin/Cisplatin +Gemcitabine/Docetaxel  /Paclitaxel/liposomes Paclitaxel /Paclitaxel polymer micelles  OR  Nedaplatin + Docetaxel | PD-L1expression＜1% |
| **Chemotherapy** | PS=2 | - NCCN:   ①***Preferred:**Carboplatin+Pemetrexed  ②***Other Recommended:**  Carboplatin+Albumin-bound paclitaxel/  Paclitaxel//Docetaxel/Etoposide/Gemcitabine  ③***Useful in Certain Circumstances:**  Albumin-boundPaclitaxel/Docetaxel/  Gemcitabine/Paclitaxel/Pemetrexed  OR  Gemcitabine+Docetaxel/Vinorelbine   - CSCO:   Gemcitabine/Paclitaxel/Vinorelbine/Docetaxel/Pemetrexed | - NCCN:   ①***Preferred:** Carboplatin+Albumin-bound paclitaxel/Gemcitabine/Paclitaxel  ②***Other Recommended:**  Carboplatin+Docetaxel/Etoposide  ③***Useful in Certain Circumstances:**  Albumin-bound paclitaxel/Docetaxel/  Gemcitabine/Paclitaxel  OR  Gemcitabine+Docetaxel/Vinorelbine   - CSCO:   Gemcitabine/Paclitaxel/Vinorelbine/Docetaxel | PD-L1expression＜1% |
| **Targeted therapy** | —— | **EGFR Mutation:** Gefitinib; Erlotinib; Icotinib; Afatinib; Dacomitinib; Osimertinib; Almonertinib; Furmonertinib  **KRAS G12C Mutation**: Treatment based on first-line regimens for non-driver genes  **ALK Rearrangement:** Lorlatinib; Ensartinib; Alectinib; Ceritinib; Brigatinib; Crizotinib  **ROS1 Rearrangement:** Crizotinib；Entrectinib；**^a^**Repotrectinib  **BRAF V600E Mutation:** Dabrafenib+Trametinib  ^a^MET Exon 14 Skipping Mutation: Capmatinib(Preferred); Tepotinib(Preferred); Crizotinib  **RET Rearrangement:** Selpercatinib(Preferred); **^a^**Pralsetinib; **^a^**Cabozantinib  **ERBB2 (HER2) Mutation:** Treatment based on first-line regimens for non-driver genes | | —— |
| **Immunotherapy** | **^a^**PS=0~2  **^b^**PS=0~1 | Atezolizumab | | PD-L1expression≥50% |
|  | **^a^**PS=0~2  **^b^**PS=0~1 | Pembrolizumab | | PD-L1expression≥50%，  *PD-L1expression≥1-49% |
|  | **^a^**PS=0~2 | **^a^**①**Preferred:**Cemiplimab-rwlc  **^a^**②**Useful in Certain Circumstances:**Nivolumab + Ipilimumab | | **^a^**PD-L1expression≥50% |
|  | **^a^**PS=0~2 | **^a^Other Recommended:** Nivolumab + Ipilimumab | | **^a^**PD-L1expression≥1-49% |
|  | **^a^**PS=0~1 | **^a^Other Recommended: ***Nivolumab + Ipilimumab | | **^a^**PD-L1expression＜1% |
| **Chemotherapy& Immunotherapy** | **^a^**PS=0~2 | **^a^**①**Preferred:**  (Carboplatin or Cisplatin) + Pemetrexed +  Pembrolizumab  OR  Cemiplimab-rwlc + Pemetrexed + (Carboplatin or Cisplatin)  **^a^**②**Other Recommended:**  Carboplatin + Albumin-bound paclitaxel +  Atezolizumab  OR  Nivolumab + Ipilimumab + Pemetrexed + (Carboplatin or Cisplatin)  OR  Cemiplimab-rwlc + Paclitaxel + (Carboplatin or Cisplatin)  OR  *Tremelimumab-actl + Durvalumab + Carboplatin + Albumin-bound paclitaxel  OR  *Tremelimumab-actl + Durvalumab + (Carboplatin or Cisplatin) + Pemetrexed | **^a^**①**Preferred:**  Carboplatin + (Paclitaxel or Albumin-bound paclitaxel) + Pembrolizumab  OR  Cemiplimab-rwlc + Paclitaxel + (Carboplatin or Cisplatin)  **^a^**②**Other Recommended:**  Nivolumab + Ipilimumab + Paclitaxel +  Carboplatin  OR  *Tremelimumab-actl + Durvalumab + Carboplatin + Albumin-bound paclitaxel  OR  *Tremelimumab-actl + Durvalumab + (Carboplatin or Cisplatin) + Gemcitabine | **^a^**PD-L1expression≥50% |
| **Chemotherapy& Immunotherapy** | **^a^**PS=0~2 | **^a^**①**Preferred:**  (Carboplatin or Cisplatin) + Pemetrexed + Pembrolizumab  OR  Cemiplimab-rwlc + Pemetrexed + (Carboplatin or Cisplatin)  **^a^**②**Other Recommended:**  *Carboplatin + Albumin-bound paclitaxel + Atezolizumab  OR  Nivolumab + Ipilimumab + Pemetrexed + (Carboplatin or Cisplatin)  OR  Cemiplimab-rwlc + Paclitaxel + (Carboplatin or Cisplatin)  OR  Tremelimumab-actl + Durvalumab +Carboplatin + Albumin-bound paclitaxel  OR  Tremelimumab-actl + Durvalumab + (Carboplatin or Cisplatin) + Pemetrexed | **^a^**①**Preferred:**  Carboplatin + (Paclitaxel or Albumin-bound paclitaxel) + Pembrolizumab  OR  Cemiplimab-rwlc + Paclitaxel + (Carboplatin or Cisplatin)  **^a^**②**Other Recommended:**  Nivolumab + Ipilimumab + Paclitaxel + Carboplatin  OR  *Tremelimumab-actl + Durvalumab + Carboplatin + Albumin-bound paclitaxel  OR  Tremelimumab-actl + Durvalumab + (Carboplatin or Cisplatin) + Gemcitabine | **^a^**PD-L1expression≥1-49% |
| **Chemotherapy& Immunotherapy** | PS=0~1 | - NCCN:   ①**Preferred:**  Carboplatin/Cisplatin+Pemetrexed + Pembrolizumab  OR  Cemiplimab-rwlc+Pemetrexed+(Carboplatin or  Cisplatin)  ②**Other Recommended:**  Nivolumab+Ipilimumab+Pemetrexed+  (Carboplatin or Cisplatin)  OR  Tremelimumab-actl+Durvalumab+Carboplatin+albumin-bound Paclitaxel  OR  Cemiplimab-rwlc+Paclitaxel+(Carboplatin or Cisplatin)  OR  *Tremelimumab-actl+Durvalumab+  (Carboplatin or Cisplatin)+Pemetrexed  OR  *Atezolizumab+Carboplatin+Albumin-bound paclitaxel   - CSCO:   Pemetrexed+Carboplatin/Cisplatin  +Pembrolizumab/Carilizumab/Sintilimab/  Tislelizumab/Atezolizumab/Sugemalimab/  Toripalimab | - NCCN   ①**Preferred:**  Carboplatin+Paclitaxel/Albumin-bound paclitaxel+Pembrolizumab  OR  Cemiplimab-rwlc+Paclitaxel+ (Carboplatin or Cisplatin)  ②**Other Recommended:**  Nivolumab+Ipilimumab+Paclitaxel+  Carboplatin  OR  Tremelimumab-actl+Durvalumab+  Carboplatin+Albumin-bound paclitaxel  OR  Tremelimumab-actl+Durvalumab+  (Carboplatin or Cisplatin) +Gemcitabine   - CSCO：   Paclitaxel/Albumin-bound paclitaxel  +Carboplatin/Cisplatin+Pembrolizumab/  Tislelizumab  OR  Paclitaxel+Carboplatin+Carilizumab/  Sugemalimab/Penpulimab  OR  Gemcitabine+Carboplatin/Cisplatin  +Sintilimab  OR  Albumin-bound paclitaxel+Carboplatin  /Cisplatin+Serplulimab | PD-L1expression＜1% |
| **Chemotherapy&**  **Anti-angiogenesis therapy** | PS=0~1 | - NCCN：   **Contraindications to PD-1 /L1inhibitors:**  Carboplatin+Paclitaxel+Bevacizumab  OR  *Carboplatin+Pemetrexed+Bevacizumab  *Cisplatin+Pemetrexed+Bevacizumab   - CSCO：   Bevacizumab in combination with Carboplatin  /Cisplatin + Bevacizumab maintenance therapy | —— | PD-L1expression＜1% |
| **^c^Combination therapy** | **^a^**PS=0~2 | **^a^Other Recommended:**  Atezolizumab+Carboplatin+Paclitaxel+  Bevacizumab | —— | **^a^**PD-L1expression≥50% |
|  | **^a^**PS=0~2 | **^a^Other Recommended:**  Atezolizumab+Carboplatin + Paclitaxel + Bevacizumab | —— | **^a^**PD-L1expression≥1-49% |
|  | **^a^**PS=0~1 | **^a^Other Recommended:**  Atezolizumab+Carboplatin + Paclitaxel + Bevacizumab | —— | **^a^**PD-L1expression＜1% |

1.The above information refers to the National Comprehensive Cancer Network (NCCN)Non-Small Cell Lung Cancer Clinical Practice Guidelines (2024.v1)

and the Chinese Society of Clinical Oncology (CSCO) Non-Small Cell Lung Cancer Diagnosis and Treatment Guidelines (2023 edition);

2.Performance Status (PS), an indicator measuring a patient's ability to maintain normal bodily functions in a non-resting state, categorized into levels 0 to 5;

3. “*” represents category II recommendations; unmarked recommendations are category I;

4. “a” represents NCCN guidelines; “b" represents CSCO guidelines, and if not specifically indicated, both guidelines are recommended consistently;

5.”c”: combination therapy refers to Anti-angiogenesis therapy & Chemotherapy & Immunotherapy.
